# Herpes simplex virus-1 fluidizes the nucleus enabling condensate formation

**DOI:** 10.1101/2025.06.20.660750

**Published:** 2025-06-21

**Authors:** Nora L Herzog, Gururaj R Kidiyoor, Sarah Keegan, Farah Korchi, David M. Chenoweth, Huaiying Zhang, Ian Mohr, Angus Wilson, Liam J Holt

**Affiliations:** 1 Institute for Systems Genetics, New York University Langone Health, 435 E 30th Street, New York NY 10016, USA; 2 Department of Microbiology, New York University School of Medicine, New York, NY 10016, USA; 3 Université Paris Cité, Magistère Européen de Génétique, 85 Boulevard Saint-Germain 75006 Paris, France; 4 University of Pennsylvania, Department of Chemistry 231 S. 34 Street Philadelphia, PA 19104-6323, USA; 5 Department of Biological Sciences, Carnegie Mellon University, Pittsburgh, PA 15213-2617, USA; 6 Department of Biochemistry and Molecular Pharmacology, New York University Langone Health, 435 E 30th Street, New York NY 10016, USA

## Abstract

Molecular processes are profoundly influenced by the biophysical properties of the cell interior. However, the mechanisms that control these physical properties, and the processes they impact remain poorly understood, especially in the nucleus. We hypothesized that some viruses might change the biophysical properties of the nucleus to favor virus survival and replication and found that herpes simplex virus 1 (HSV-1) increases the mesoscale fluidity of the nucleus. The HSV-1 protein ICP4 caused fluidization and enabled growth of synthetic nuclear condensates. Conversely, conditions that decreased nuclear fluidity inhibited the formation of viral replication compartment condensates and reduced infectious virus production. Together, our data suggest that ICP4 increases nuclear fluidity to promote the formation of condensates that drive the progression of the HSV-1 life cycle. We speculate that a key function of ICP4 is to overcome the crowding and elastic confinement within cell nuclei that are a fundamental barrier to virus replication.

## INTRODUCTION

The biophysical properties of the cell interior have a profound impact on biochemistry and cellular organization. The fluidity (the inverse of viscosity) of the cell interior is determined by multiple factors, including macromolecular crowding and confinement within elastic structures, which together decrease motion^1,2^, and non-thermal energy from metabolic and motor activities, which increases motion^3^. The effective fluidity of the cell interior decreases in a non-linear fashion as particle size increases^4^, greatly hindering the motion of mesoscale (50–1000 nanometer length-scale) particles and cellular structures^5^. Indeed, cells are thought to border on a liquid-solid (jamming) transition at this scale, that is prevented by metabolic and motor activities driving increased motion^6,7^. These crowded, non-equilibrium properties are thought to be tuned to enable efficient biochemistry and cellular organization; the importance of maintaining optimal physical properties is highlighted by studies that show that excessive macromolecular crowding in the cytoplasm prevents cell growth^8^ while cytoplasmic dilution leads to senescence^9^. However, despite this fundamental importance, very little is known about the regulation of the physical properties of the nucleus.

The formation of biomolecular condensates has emerged as an important principle for biological organization in recent years^10^. Condensates are mesoscale structures, and their formation can be strongly influenced by the physical environment^11^. Condensates often form through a nucleation and growth mechanism where macromolecular crowding favors nucleation but inhibits growth, while increased non-thermal energy facilitates coalescence by reducing elastic confinement and increasing the motion of condensates, thus enabling fusion^12,13^. In addition, chromatin fibers have been shown to mechanically arrest condensate growth by restricting their growth and diffusion, highlighting the importance of nuclear biophysical properties for nuclear condensate formation^14,15^.

Viruses are obligate intracellular parasites that manipulate host cells to drive viral replication. Indeed, viruses can drive extreme perturbations of cellular physiology including suppression of host cell transcription^16,17^, translation, and mRNA splicing^18^, as well as changes to chromatin architecture^19–21^ and modifications to cellular histones^22–24^. Therefore, we hypothesized that some viruses might alter the biophysical properties of host cells to either globally disfavor host biochemistry and/or favor viral processes. Diverse viral families form condensates as part of their life cycle, including rabies virus (RABV)^25^, vesicular stomatitis virus (VSV)^26^, and severe acute respiratory syndrome coronavirus 2 (SARS-CoV-2)^27^. This led us to hypothesize that viruses might alter host cell physical properties to facilitate condensate assembly. Herpes simplex virus 1 (HSV-1) is a double-stranded DNA virus that replicates within viral condensates in the nucleus and alters host chromatin^28,29^. Therefore, we focused our studies on this virus that infects approximately two thirds of the world’s population^30^, leading to cold sores, encephalitis, as well as lasting neurological disability in the case of neonatal herpes^31^.

The HSV-1 life cycle consists of three phases: immediate early (IE), during which the first genes are transcribed, early (E), during which proteins required for viral DNA synthesis are produced; and late (L), which occurs after viral DNA replication has begun ^32^. During the early and late phases of the HSV-1 life cycle interchromatin domains are enlarged, enabling more efficient egress of virus capsids^33^. HSV-1 also marginalizes and condenses chromatin late in infection, in a process known as rearrangement of cellular chromatin (ROCC)^19,29^. Previous studies showed that viral replication compartments (vRCs) initially nucleate as small pre-RCs and then coalesce into larger vRC condensates^34,35^. Host DNA is excluded from vRCs during HSV-1 infection, suggesting that cellular chromatin must be manipulated for vRCs to form^20^. Studies examining the relationship between cellular chromatin architecture and vRC formation in HSV-1 infection correlated chromatin rearrangement with vRC expansion^29^. Taken together, this suggests that ROCC may facilitate the formation of vRCs^36^. These studies showed that virus-directed processes including DNA synthesis, virus assembly, and virus egress may drive nuclear remodeling on the micron (organelle) scale late in viral infection. However, very little is known about the effect of HSV-1 on the biophysical properties of the nucleus at early times of infection, especially at the mesoscale.

Mesoscale fluidity can be studied by microrheology, which is the inference of the biophysical properties of materials through analysis of the motion of tracer particles. We developed nuclear Genetically Encoded Multimeric nanoparticles (nucGEMs) to enable high throughput microrheology in the nucleus^37^. We found that HSV-1 infection increased mesoscale diffusivity at early time points of infection through a mechanism that is independent of previously described changes in nuclear architecture. Furthermore, we found that the IE protein ICP4 was sufficient to increase mesoscale nuclear diffusivity. We showed that the ICP4-driven nuclear fluidization can facilitate condensate growth, and that inhibition of mesoscale fluidization inhibited vRC condensate maturation and led to a decrease in infectious titer. In conclusion, we determined that HSV-1 fluidizes the infected cell nucleus, thus priming the nuclear microenvironment for optimal maturation of vRCs.

## RESULTS

### HSV-1 infection alters the biophysical properties of the nucleus.

**Figure 1.**
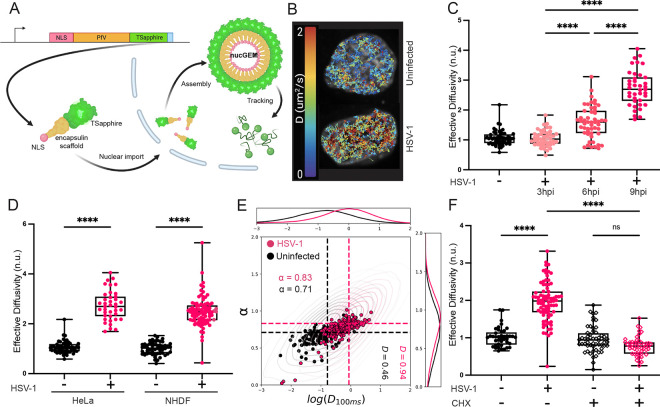
Nuclear diffusivity increases during HSV-1 infection and requires new protein synthesis. A) Schematic of the nucGEM system. The nucGEM transgene encodes a *Pyrococcus furiosus* encapsulin (PfV) scaffold fused to an N-terminal nuclear localization signal (NLS) and a C-terminal T-Sapphire fluorophore. Upon nuclear import, nucGEM monomers assemble into a 40 nm diameter fluorescent nanoparticle. (B) Tracks of individual nucGEMs from a representative infected and uninfected HeLa cell and color-coded according to the effective diffusion coefficient. (C) Effective diffusivity of nucGEMs in uninfected HeLa cells or HSV-1 infected cells at 3 hpi, 6 hpi, and 9 hpi. Infections were with wild type HSV-1 (strain KOS) at an MOI of 5. Cells were imaged at 100 Hz, and nucGEM particles were tracked. Individual data points represent the median effective diffusion of all tracks in one cell. Statistical comparisons were performed using a Kruskal-Wallis test. n>136; N≥4 biological replicates. (D) Comparison of the median effective diffusivity in HeLa cells and normal human dermal fibroblasts (NHDFs) with or without HSV-1 infection at 9 hpi. Statistical comparisons were performed using a two-way ANOVA. n>136; N≥3 biological replicates. (E) Graphical representation of the anomalous exponent (a) compared to the log effective diffusion (*log(D*_*100ms*_*)*) of tracks in HSV-1 infected (pink) or uninfected (dark gray) cells from three representative experiments. Frequency maps along each axis represent the number of cells with a given value. Statistical analysis available in supplemental table 1. (F) NHDFs expressing nucGEMs were pretreated for 30 mins with cycloheximide (CHX) and where indicated, infected with HSV-1 at an MOI of 5 and imaged at 9 hpi. Statistical comparisons were performed using a two-way ANOVA. n>82; N≥3 biological replicates. **** signifies p<0.0001.

Until recently, microrheology has been labor intensive and slow due to difficulties in introducing tracer particles, especially to the nucleus. To overcome these limitations, we developed nuclear Genetically Encoded Multimeric nanoparticles (nucGEMs, Fig. 1A). nucGEMs consist of a fusion between a self-assembling protein (the *Pyrococcus furiosus* encapsulin protein)^38^ to the mSapphire fluorescent protein^39^. We used the SV40 nuclear localization signal to target these monomers to self-assemble into a bright 40 nm particle in the nucleus^37^. The protein monomer-encoding transgene was integrated into the genome by lentiviral transduction, leading to constitutive expression of nucGEMs (Fig. 1A)^39^. Single particle tracking of nucGEMs allows calculation of an effective diffusivity at the 40 nm length-scale and 100 ms time-scale, which reflects the effective mesoscale fluidity of the nucleus. The effective diffusivity at this length and time-scale is strongly impacted by macromolecular crowding, confinement, and non-thermal energy^6^.

To characterize the effects of herpes simplex virus (HSV-1) infection on nuclear fluidity, we expressed 40 nm diameter nuclear GEMs (nucGEMs) in HeLa cells^37^ and infected them with HSV-1 at a multiplicity of infection of 5 (MOI = 5). Cells were imaged by spinning disk confocal microscopy at 100 Hz, and single particle tracking was performed to determine the effective diffusivity at the 100 ms timescale (D_eff_100ms_, Fig. 1A, Fig. 1B). We observed that the effective diffusivity of nucGEMs increased over the course of infection, starting as early as 6 hpi (Fig. 1C). We next examined normal human dermal fibroblasts (NHDFs) to determine if this increase was also seen in primary cells. Cells were infected with HSV-1 at MOI=5 as before and a similar increase in nucGEM diffusivity was observed (Fig. 1D). Since both NHDFs and HeLa cells showed an increase in D_eff_100ms_, subsequent virus infection experiments were conducted in NHDFs as the more physiologically relevant cell line for HSV-1 lytic infection, and key experiments were also confirmed in HeLa cells to assess generality (Supplemental Fig. 1, Supplemental Fig. 2). To further characterize the nature of the changes in nucGEM motion, we graphed the log of effective diffusivity (D_eff_100ms_) versus the anomalous exponent (**α**) (Fig. 1E). Shifts in **α** are indicative of changes to the type of motion—for example due to directed motion, or binding interactions with the environment—while shifts in D_eff_100ms_ indicate changes in the effective fluidity^40,41^. The anomalous exponent was relatively unaffected by HSV-1 infection, while D_eff_100ms_ increased, indicating the increased diffusivity of nucGEMs is mostly due to increased nuclear fluidity.

### HSV-1 transcription and translation are required to increase nucGEM diffusivity.

To determine if HSV-1 tegument or gene expression was required to increase nucGEM diffusivity, we infected cells with HSV-1 inactivated by UV. The tegument is a layer of proteins located inside the viral envelope, but surrounding the viral capsid^28^. UV-inactivation prevents viral transcription, but intact tegument, and capsid can still enter the host cell. Virus-encoded gene products were not detected in cells infected with UV-inactivated HSV-1, confirming lack of transcription, and nuclear diffusion did not detectably increase (Supplemental Fig. 1A, B, C). Furthermore, increased nuclear diffusion was not observed in HSV-1 infected cells treated with the translation inhibitor cycloheximide (CHX), which allows virus immediate early (IE) RNA accumulation but inhibits new protein synthesis (Fig. 1F, Supplemental Fig. 1D, E). Taken together, these data show the increase in nucGEM diffusivity is dependent on both virus gene transcription and new protein synthesis.

### Fluidization is independent of viral DNA synthesis, chromatin margination, and nuclear volume changes.

**Figure 2.**
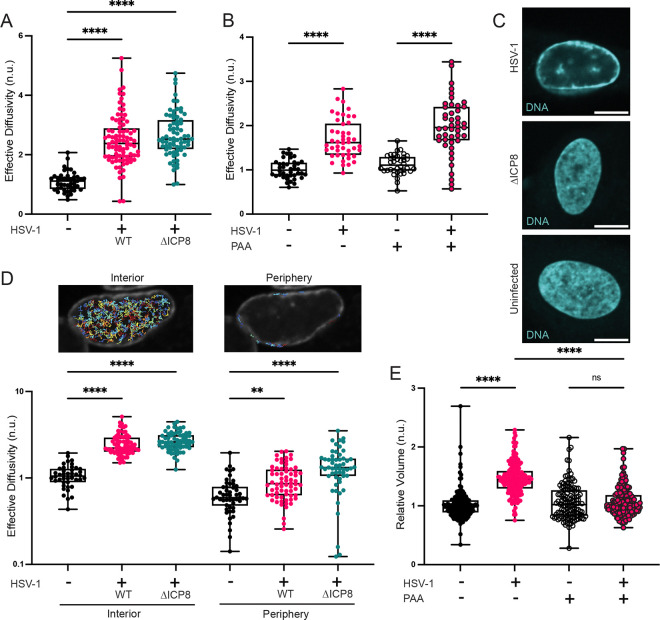
HSV-1 infection fluidizes the nucleus independent of viral DNA synthesis, chromatin margination, and nuclear volume changes. (A) NHDFs expressing nucGEMs were infected with WT HSV-1 or HSV-1 ΔICP8 at an MOI of 5. At 9 hpi, GEM movies and nuclei images were obtained and analyzed to determine effective diffusivity as described (Methods). Statistical comparisons were performed using a Kruskal-Wallis test. n>83; N≥3 biological replicates. (B) NHDFs were treated with 300 μg/mL phosphonoacetic acid (PAA) and either left uninfected or infected with WT HSV-1. Data was obtained as in panel A. Statistical comparisons were performed using a two-way ANOVA. n>86; N≥3 biological replicates. (C) Cells were stained using vital stain SiR-DNA, which binds to AT-rich DNA, to visualize the host nucleus. Samples were obtained at 9 hpi in uninfected, ΔICP8 HSV-1, and WT HSV-1 infected cells (scale bar, 10 μm). (D) nucGEM diffusivity in the periphery and the interior of the cells during WT HSV-1 and ΔICP8 HSV-1 infection compared to uninfected cells. Periphery is defined as an 1830 nm band, the approximate size of marginated chromatin in WT HSV-1 infection, around the perimeter of the nucleus. Statistical comparisons were performed using a two-way ANOVA. n>80; N≥3 biological replicates. (E) Relative nuclear volume of cells from experiments in (C), calculated with Foci-Counting. Statistical comparisons were performed using a two-way ANOVA. All volumes normalized to the median uninfected nuclear volume by experiment. n>249; N≥2 biological replicates. IF images are representative of N=3 biological replicates. ** signifies p<0.01, **** signifies p<0.0001.

HSV-1 DNA replication is linked to architectural rearrangement of the infected nucleus, most notably, the condensation and margination of cellular chromatin^19,20,29,36^. Previously observed changes in nuclear biophysical properties during HSV-1 infection examined changes in diffusivity of virus capsids, highlighting effects that were temporally linked to these large-scale rearrangements^29,33,42^. Therefore, we asked if the observed increase in nucGEM diffusivity required virus DNA replication. We blocked virus DNA replication either genetically by using a mutant virus deficient for the essential single-stranded DNA binding protein ICP8 (d301, henceforth referred to as ΔICP8)^43^, or by inhibiting the viral DNA polymerase with phosphonoacetic acid (PAA)^44^. Unexpectedly, nucGEM diffusivity increased even when virus DNA replication was prevented or inhibited (Fig. 2A, Fig. 2B, Supplemental Fig. 2A). Furthermore, when DNA replication was blocked by infection with ΔICP8 or by PAA treatment, chromatin margination was not observed, indicating that the mesoscale fluidization of the nucleus was independent of these large-scale rearrangements (Fig. 2C).

We next explored spatial regulation of nuclear properties. Chromatin in the periphery of the nucleus tends to be more compact than that in the center^42^. Therefore, we tested whether nucGEM motion was distinct at the periphery compared to the interior of the nucleus. We compared nucGEM motion upon wild-type (WT) HSV-1 or ΔICP8 infection in an outer ring of 1800 nm width at the periphery of the nucleus (corresponding to the approximate position of marginated chromatin in NHDFs during WT HSV-1 infection) to that in the region of the nucleus interior to this ring. Diffusivity was lower in the periphery than the interior for all conditions, but both compartments were fluidized to a similar extent during WT and ΔICP8 infection (Fig. 2D).

Finally, we estimated nuclear volume in the absence of virus DNA replication. Nuclear volume is known to increase during HSV-1 infection and increased volume could reduce macromolecular crowding^45^. Our results showed that while nuclear volume does increase during WT HSV-1 infection, it does not change detectably during ΔICP8 infection or when DNA replication was inhibited with PAA treatment (Fig. 2E). This indicates nuclear fluidization during early HSV-1 infection does not require increased nuclear volume.

### The HSV-1 immediate early protein ICP4 is required to fluidize the infected cell nucleus.

**Figure 3.**
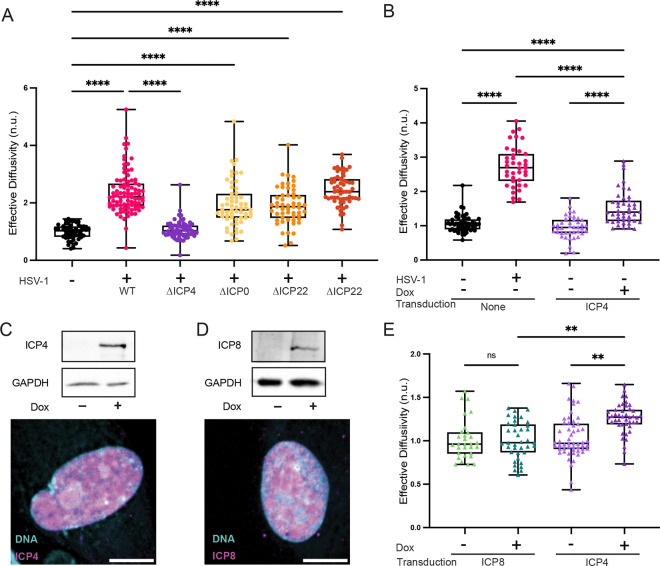
HSV-1 immediate early protein ICP4 is necessary to fluidize the infected cell nucleus and sufficient to fluidize the nucleus of uninfected cells. (A) NHDFs expressing nucGEMs were infected with WT KOS HSV-1, ΔICP4 HSV-1, ΔICP22 HSV-1, or ΔICP27 HSV-1 at an MOI of 5, or with ΔICP0 at an MOI of 10. At 9 hpi, GEM movies and nuclei images were obtained and analyzed to determine effective diffusivity as described (Methods). Statistical comparisons were performed using a Kruskal-Wallis test. n>108; N≥3 biological replicates. (B) HeLa cells expressing nucGEMs were transfected with a plasmid expressing codon-optimized ICP4 under a tet-promoter and induced with 3 μg/mL doxycycline for 9 h. At 9hpt, imaging data was acquired as in panel A. n>81; N≥4 biological replicates. (C) Immunoblot for ICP4 or (D) ICP8 alongside GAPDH as a loading control to verify induction in NHDFs at 9 hpt. Samples were fixed using PFA, then probed by IF for ICP4 (C) or ICP8 (D) to visualize localization in the cell (scale bar, 10 μm). (E) NHDFs expressing nucGEMs were transduced as described (Methods) with a lentivirus expressing codon-optimized ICP4 or ICP8 under a tet-promoter and induced with doxycycline for 9 h. Imaging data was acquired as in panels A and B. n>107; N≥5 biological replicates. IF images are representative of N=3 biological replicates. ** signifies p<0.01, **** signifies p<0.0001.

HSV-1 late genes require DNA replication for their expression^28^. As the virus-induced increase in nucGEM effective diffusivity was observed in the absence of virus DNA replication, we reasoned that a protein (or proteins) expressed during the immediate early or early phases of the HSV-1 life cycle might be required to increase nuclear fluidity. HSV-1 expresses four nuclear immediate early proteins during lytic infection: ICP0, ICP4, ICP22, and ICP27. We infected NHDFs with mutant viruses individually deficient in ICP0, ICP4, ICP22 or ICP27 and analyzed nucGEM diffusivity. NHDFs infected with HSV-1 mutant viruses deficient in ICP0, ICP22, or ICP27 all increased diffusivity by 9 hpi, similar to cells infected with WT virus. However, nuclear diffusivity did not increase in NHDFs infected with the ICP4-deficient virus (Fig. 3A). Viruses lacking ICP0, ICP22, or ICP27 all expressed ICP4 as measured by immunoblot (Supplemental Fig. 3A). Thus, fluidization of the nucleus in HSV-1 infected cells was dependent upon a single IE gene, ICP4.

### ICP4 is sufficient to fluidize the nucleus of uninfected cells.

To ascertain whether expression of ICP4 alone was sufficient to increase nucGEM diffusivity, we exogenously expressed ICP4 in uninfected HeLa cells by transiently transfecting cells with a plasmid containing ICP4 under a doxycycline-inducible promoter. Exogenous expression of ICP4 led to significantly lower levels of protein expression than during HSV-1 infection (Fig. 3B, Supplemental Fig. 3B). Nevertheless, even this low level of exogenous ICP4 was sufficient to increase nucGEM diffusivity in HeLa cells after 9h of induction. To enable a similar experiment in NHDF cells, which cannot be readily transfected, we next constructed lentivirus vectors for the inducible expression of either ICP4 or a control protein, ICP8, that was not associated with increased diffusivity during HSV-1 infection. The efficacy of ICP4 and ICP8 expression and their appropriate nuclear localization were verified by immunoblot and indirect immunofluorescence (IF) respectively (Fig. 3C, Fig. 3D). ICP4 induction also increased nucGEM diffusivity in primary NHDFs, while ICP8 induction had no detectable effect on nucGEM diffusivity (Fig. 3E). Thus, expression of ICP4, but not another HSV-1 nuclear protein ICP8, is sufficient to partially recapitulate the nuclear fluidization that occurs during HSV-1 lytic infection.

### Both the N-terminal and C-terminal domains of ICP4 are required to increase nucGEM diffusivity in uninfected cells.

**Figure 4.**
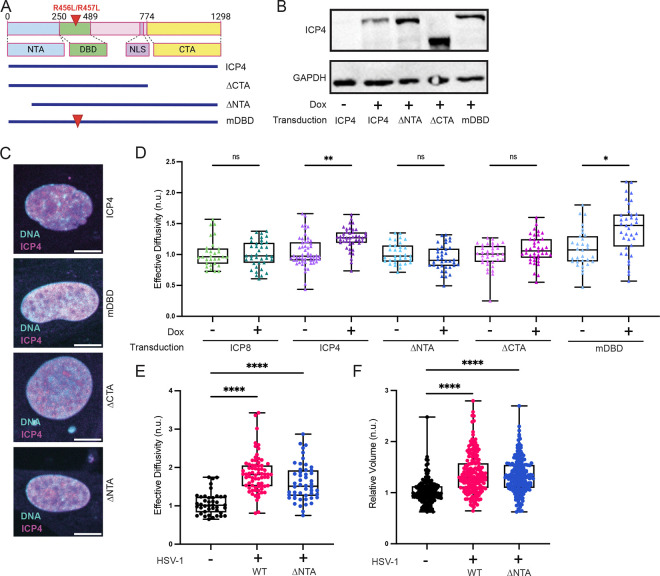
Sequences at both the N- and C-terminus of ICP4 are required to increase nucGEM diffusivity in uninfected cells. (A) Schematic showing the functional domains of ICP4 and boundaries of ICP4 mutants used in this study. Domains correspond to the DNA-binding domain (DBD), nuclear localization signal (NLS), N-terminal transactivation domain (NTA) and C-terminal transactivation domain (CTA). In addition to N- and C-terminal truncation mutants (ΔNTA and ΔCTA, respectively) we also tested double arginine to leucine substitutions at residues 456 and 457 within the DBD that disrupts sequence-specific binding (mDBD). (B) Immunoblot showing ICP4 expression for the HSV-1 mutants used in (A). (C) Images of representative lentivirus transduced cell nuclei showing indirect immunofluorescence detection of ICP4 to confirm appropriate nuclear localization (scale bar, 10 μm). (D) NHDFs expressing nucGEMs were transduced as described (Methods) with lentiviruses expressing codon-optimized ICP4, ICP8, or ICP4 mutants illustrated in (A) under a tet-promoter and induced with doxycycline for 9 h. GEM movies were acquired and the effective diffusivity determined. n>113; N≥5 biological replicates. (E) NHDFs expressing nucGEMs were infected with HSV-1 virus expressing ΔNTA isoform of ICP4 at MOI = 5. GEM movies were acquired as in panel D. n>116; N≥3 biological replicates. (F) Relative nuclear volume of cells from experiments in (E), calculated with Foci-Counting (Methods). All volumes were normalized to the median uninfected nuclear volume by experiment. n>357; N≥3 biological replicates. IF images and immunoblots are representative of N=3 biological replicates. Statistical comparisons were performed using a Kruskal-Wallis test for all data. * signifies p<0.05, ** signifies p<0.01, **** signifies p<0.0001.

Next, we used mutants to dissect which functions of ICP4 are required for nuclear fluidization. ICP4 has three known functional domains, the N-terminal activation domain (NTA), the C-terminal activation domain (CTA), and the DNA-binding domain (DBD)^46^. We engineered three mutants in the inducible ICP4 construct: a deletion of the CTA (ICP4-ΔCTA)^47^, a truncation of the first 90 amino acids of the NTA (ICP4-ΔNTA)^47^, and a double point-mutation in the DBD (ICP4-*m*DBD)^48^(Fig. 4A). This mutation in the DBD prevents specific binding of a sequence found in ICP4-regulated promoters, but does not prevent non-specific binding of ICP4 to host chromatin^46,48,49^. We induced each mutant for 9 h in uninfected NHDFs and analyzed protein expression. Immunoblotting for ICP4 indicated that all ICP4 mutant proteins were expressed at comparable levels to WT ICP4 (Fig. 4B) and their appropriate nuclear localization was verified by IF (Fig. 4C). We additionally induced each mutant for 9 h in NHDFs containing nucGEMs and analyzed nuclear diffusivity. We found that induction of ICP4-*m*DBD increased nucGEM diffusivity to levels comparable to WT ICP4 induction. However, increased nucGEM diffusivity was not detected following induction of ICP4-ΔNTA or ICP4-ΔCTA mutant proteins (Fig. 4D). We did not detect changes in nuclear volume between induced and uninduced cells (Supplemental Fig. 4A). As there is an HSV-1 mutant virus that contains the ΔNTA ICP4 isoform and produces infectious titer, we examined nucGEM diffusivity in the context of infection with this ΔNTA mutant virus. We found an increase in nucGEM diffusivity during ΔNTA mutant virus infection (Fig. 4E), indicating that this domain is not required for fluidization in the presence of other virus activities. The ΔNTA mutant virus drove an increase in nuclear volume (Fig. 4F). This may suggest an additional ICP4-independent mechanism for nuclear fluidization related to nuclear volume increase in the context of full virus infection. Together, these results suggest that nuclear fluidization by ICP4 does not require specific DNA binding, but does require the C-terminal and N-terminal activation domains in uninfected cells.

### ICP4 induction does not alter global transcription.

The biophysical properties of the nucleus can be impacted by a wide variety of factors. For example, transcription increases non-thermal energy but also increases crowding by increasing RNA abundance. While ICP4 is an essential transcription factor for HSV-1, it also binds promiscuously to the host genome through early phases of HSV-1 infection^50^. To test if ICP4 induction impacts global transcription rates when exogenously expressed, we incubated cells for 2 h with the nucleoside analog 5-ethynyl uridine (5EU). 5EU is incorporated into newly synthesized RNA and can subsequently be visualized by fixing cells and using click chemistry to conjugate its alkyne group to an azide-modified fluorescent dye^51,52^. The intensity of fluorescence is proportional to the total amount of RNA produced during the pulse of 5EU incubation. We performed 5EU labeling with or without exogenous expression of ICP4, ICP8, ICP4-ΔNTA, and ICP4-ΔCTA for 7 h. Our data showed no changes in 5EU incorporation during ICP4 induction compared to its uninduced controls (Supplemental Fig. 4B). ICP8 induction, ΔNTA induction, and ΔCTA induction also did not change global transcription as measured by 5EU (Supplemental Fig. 4B). Thus, nuclear fluidization by exogenous ICP4 is unlikely to be due to global changes in host cell transcription.

### ICP4 induction increases motion of bound histones

**Figure 5.**
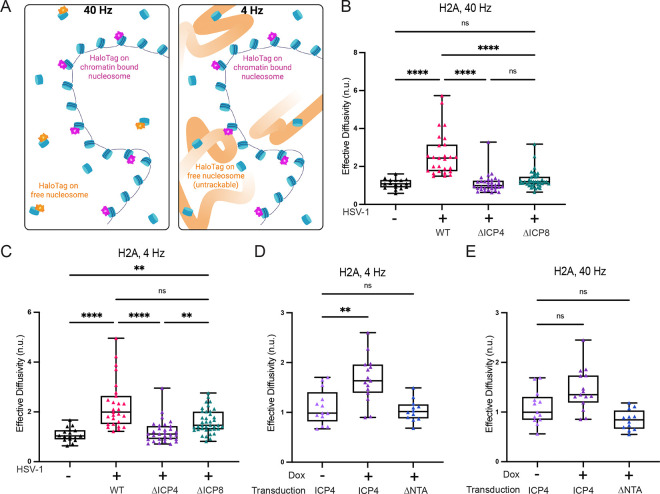
Movement of bound histones increases during ICP4 induction and during HSV-1 infection dependent on the presence of ICP4. (A) Schematic showing single-particle tracking of tagged histones at short (40 Hz) and long (4 Hz) time scales. Orange particles represent freely diffusing histones, which are only visible at the 40 Hz time scale. Purple particles represent bound histones, which are visible at 40 Hz and 4 Hz time scales. (B, C) NHDFs expressing a HaloTag-histone H2A fusion were infected with HSV-1 wild-type, ΔICP4, or ΔICP8 and single molecule tracking was performed at 9 hpi to capture freely diffusing histones (40 Hz, panel B) and bound histones (4 Hz, panel C). n>45; N≥3 biological replicates. (D, E) NHDFs expressing Halo-H2A were transduced with lentiviruses expressing either WT or ΔNTA ICP4. After addition of doxycycline for 9 h to induce ICP4 expression, single molecule tracking was performed at 4 Hz (D) and 40 Hz (E) time scales. n>35; N≥3 biological replicates. Statistical comparisons were performed using a Kruskal-Wallis test for all data. ** signifies p<0.01, **** signifies p<0.0001.

The biophysical properties of the nucleus are also affected by chromatin. For example, confinement, porosity, and non-thermal motion can all be affected by chromatin organization and active processes such as chromatin remodeling^53–55^. Previous work on ICP4 showed that it co-purifies with the chromatin remodeling complexes SWI/SNF, INO80, and NuRD^56^. To determine if increased chromatin dynamics could explain the ICP4-driven increase in nucGEM diffusivity, we transduced primary NHDFs with a HaloTag-fused histone 2A (HaloTag-H2A) construct. HaloTag is a self-labeling tag that can bind covalently to bright fluorescent HaloTag Ligands (HTLs) enabling single molecule tracking^57^. We used highly inclined and laminated optical sheet (HILO) microscopy of HaloTag-H2A bound to JF646 HTL to perform single particle tracking and determined the effective diffusivity at short (250 ms) or longer (2500 ms) timescales. We used a 40 Hz frame rate to analyze H2A motion at short (250 ms) timescales. At this frame rate both chromatin bound and unbound H2A can be tracked. To analyze motion at longer (2500 ms) timescales, we reduced our time resolution to a 4 Hz frame rate (see methods). At this lower time resolution, rapidly diffusing, unbound Halotag-H2A is blurred by motion and cannot be detected, and only bound histones are analyzed (Fig. 5A, Supplemental Fig. 5A).

At 40 Hz, we found that HaloTag-H2A diffusivity increased during WT HSV-1 infection, but not during ΔICP4 or ΔICP8 infection (Fig. 5B). However, when we imaged at 4 Hz to preferentially analyze the motion of chromatin-bound HaloTag-H2A, we saw that both WT HSV-1 and ΔICP8 infection increased diffusivity (Fig. 5C). Thus, motion of bound histones correlates to the increased mesoscale diffusivity of nucGEMs, as both increase during HSV-1 infection in the absence of virus DNA replication.

We next assessed whether ICP4 induction alone was sufficient to increase the motion of HaloTag-H2A. We found that ICP4 induction is sufficient to increase the motion of bound H2A (Fig. 5D, Supplemental Fig. 5B), but this change was not apparent when analyzing both bound and free H2A at faster time scales (Fig. 5E, Supplemental Fig. 5C). We also found that ICP4-ΔNTA expression did not detectably increase HaloTag-H2A motion on either timescale, further correlating our histone tracking with our data on nucGEM diffusivity (Fig. 5D, Fig. 5E). These results are consistent with the model that ICP4 expression fluidizes the nucleus by increasing chromatin motion.

### High cell confluency and osmotic compression attenuate nuclear fluidization.

**Figure 6.**
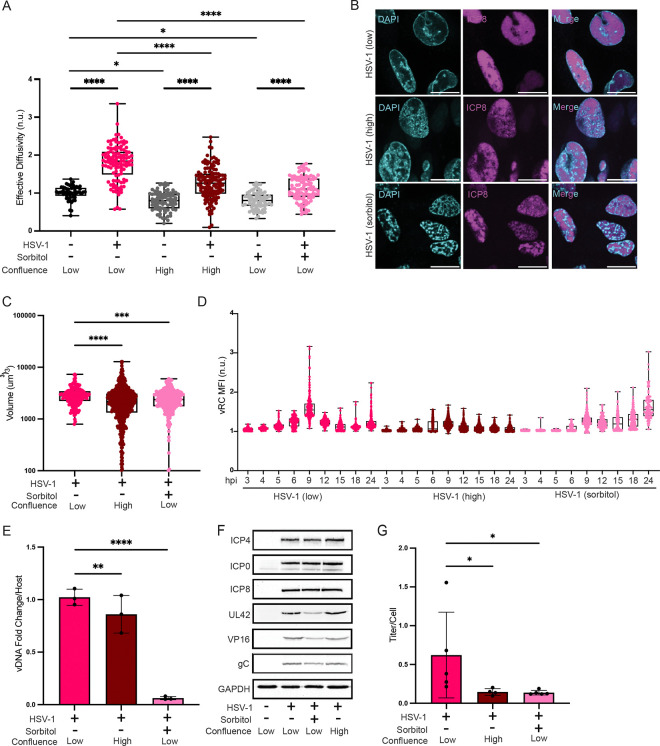
Countering nuclear fluidization disrupts viral replication compartment formation and decreases infectious virus production. (A) Nuclear crowding was accentuated by growing cells to confluence or by raising osmotic pressure using sorbitol. NHDFs expressing nucGEMs were grown into either 70% confluency (low) or to full confluency (high) and infected with HSV-1 WT at MOI = 5. At 2.5 hpi, cells were left untreated or were treated with 150 mM sorbitol. nucGEM diffusivity was measured at 9 hpi and statistical comparisons performed using a two-way ANOVA with Bonferroni’s correction. n>136; N≥3 biological replicates. (B) At 9 hpi HSV-1 WT infected cells were fixed using PFA and processed for indirect immunofluorescence microscopy to detect ICP8, a marker of the viral replication compartments (vRCs) (scale bar, 20 μm). (C) Total vRC volume from cells in (B) was quantified with Foci-Counting using a Z-stack of the nucleus. Statistical comparisons were performed using a one-way ANOVA. n>784; N≥4 biological replicates. (D) NHDFs were treated as in panel A and fixed using PFA for immunofluorescence as in panel B at the indicated time points. Mean fluorescence intensity (MFI) for ICP8 indirect immunofluorescence detection within the nucleus was calculated using Foci-Counting and normalized to background. Statistical analysis available in supplemental table 2. n>305; N≥3 biological replicates. (E) Total DNA was collected at 9 hpi and analyzed by qPCR to determine relative HSV-1 genomic DNA copy number. Using a gene cassette standard for HSV-1 UL36 DNA, total copies of viral DNA were quantified. Counts were normalized to a cellular locus and fold change over HSV-1 (low) was calculated to compare viral DNA replication in HSV-1 (high) and HSV-1 (sorbitol) to HSV-1 (low). Statistical comparisons were performed using a linear mixed-effects model. N≥3 biological replicates. (F) Immunoblot for a representative panel of IE, E, and L HSV-1 proteins in HSV-1 (high) and HSV-1 (sorbitol) compared to HSV-1 (low). (G) Supernatant was collected at 9 hpi and quantified by plaque assay to determine the infectious titer per cell. Statistical comparisons were performed using a one-way ANOVA. N≥4 biological replicates. IF images are representative of N=3 biological replicates. * signifies p<0.05, ** signifies p<0.01, *** signifies p<0.001, **** signifies p<0.0001.

To ascertain the importance of nuclear fluidization for HSV-1 infection, we used two orthogonal approaches to alter the biophysical properties of the nucleus. First, we grew cells to high confluence. We previously found that, upon contact inhibition, epithelial cells stop growing but undergo an additional cell division resulting in decreased cell and nuclear volume^58^. We confirmed that high confluence also decreased nuclear volume in NHDFs (Supplemental Fig. 6A). Decreased nuclear volume is predicted to increase the concentration of macromolecules and chromatin. Consistent with this prediction, we found that nucGEM diffusivity was significantly decreased in these highly confluent cells (Supplemental Fig. 6B). Furthermore, the increase in nucGEM diffusivity during HSV-1 infection was attenuated in these conditions (Fig. 6A). As an orthogonal approach to decrease nucGEM diffusivity, we used osmotic compression. We determined that 150mM sorbitol was sufficient to decrease nucGEM diffusivity in NHDFs such that the increase in nuclear fluidity at 9 hpi was mostly prevented (Fig 6A, Supplemental Fig. 6C, 6D).

### Preventing nuclear fluidization disrupts viral replication compartment maturation and decreases infectious virus production.

The formation of viral replication compartments (vRCs) is a key step in progression of the HSV-1 life cycle^59,60^. Formation of vRCs has been shown to proceed via formation of pre-vRCs and their subsequent coalescence into vRCs^61,62^. Since cytoplasmic fluidity was previously shown to affect the formation of cytoplasmic biomolecular condensates^13,63^, we hypothesized that fluidization of the nucleus would be important for vRC coalescence.

To test this idea, we infected cells with HSV-1 in control, high confluence, and sorbitol conditions, and probed for ICP8, which is a marker of vRCs. Indeed, when nuclear fluidization was prevented by high confluence or sorbitol treatment, vRCs did not fully mature into a single vRC, but instead multiple smaller vRCs formed (Fig. 6B), and total vRC volume was reduced (Fig. 6C).

To determine if the differences in vRC maturation were due to delayed life cycle progression under high confluence and sorbitol conditions, we performed a time course assaying vRC maturation under all three conditions from 3 hpi to 24 hpi. While the maturation of vRCs appeared to be delayed under sorbitol treatment compared to untreated cells, vRCs did not detectably coalesce in sorbitol treated or high confluence conditions (Fig. 6D, Supplemental Fig. 6E, 6F) even at much later timepoints. These data suggest that nuclear fluidization enables vRC coalescence

To assess the function of vRCs, we measured viral DNA replication using qPCR under high confluence and sorbitol treatment compared to WT HSV-1 infection at 9 hpi. Sorbitol treatment led to a ten-fold reduction in viral DNA copy number, while high confluence led to a modest, but significant reduction of viral DNA copy number (Fig. 6E).

We also assessed the production of virus proteins. High confluence did not appear to impact the accumulation of representative virus IE, E or L proteins compared to infection of sub-confluent cells, and sorbitol treatment only partially impacted virus protein accumulation (Fig. 6F).

Finally, we collected the supernatant from each condition and performed plaque assays to calculate the infectious titer. Both high confluence and sorbitol treatment reduced viral titer by approximately three-fold and four-fold, respectively (Fig. 6G).

Together, these results are consistent with the hypothesis that nuclear fluidization enables vRC condensate formation and optimal progression of the HSV-1 life cycle.

### Fluidization of the nucleus by ICP4 facilitates growth of synthetic condensates.

**Figure 7.**
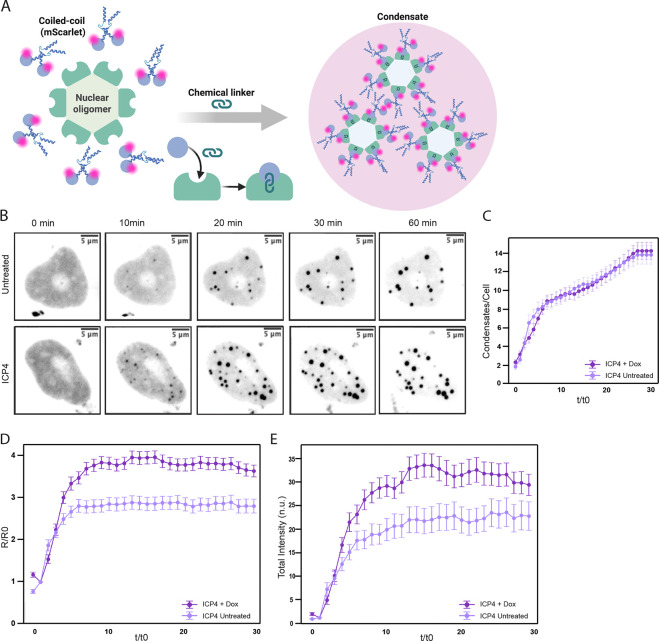
Fluidization by ICP4 facilitates artificial condensate growth. (A) Schematic of a two-component artificial condensate system that uses a fluorescently-tagged coiled-coil phase-separating protein (Mad1, blue helices) fused to E. coli dihydrofolate reductase (eDHFR, blue circles) and a nuclear hexamer (HO Tag3, light green) fused to Halo (dark green). Upon addition of chemical dimerizer Trimethoprim-fluorobenzamide-Halo ligand (TFH), eDFHR conjugates to Halo, forming condensates. Condensate protein in the diffuse or condensed phases can be visualized through mScarlet fluorescence. (B) HeLa cells were cotransfected with ICP4 expression plasmid and the two plasmids encoding the artificial condensate system as described (Methods). Cells were treated with 3 μg/mL doxycycline for 8 h or left untreated, then imaged for 1 timepoint before being treated with 50 nM TFH. Cells were imaged every 2 minutes after TFH treatment for a total of 1 h. Images shown are a max projection of the nuclear condensates at the indicated time (scale bar, 5 μm). (C) Graph showing the average number of condensates identified per cell in with (+Dox) or without (untreated) ICP4 expression. (D) Graph showing the average radii over time for condensates in cells expressing ICP4 (+Dox) compared to those without ICP4 expression (untreated). The radii were normalized to the initial droplet size (R/R0), and time was normalized to the timepoint at which condensates nucleated (t/t0). (E) Quantitation of average total intensity of each condensate over time for condensates in cells with (+Dox) or without (untreated) ICP4 expression. Total condensate intensity for each individual condensate was normalized to the mean intensity of the nucleus in the diffuse phase (pre-TFH treatment), and time was normalized to the timepoint at which condensates nucleated (t/t0). Statistical analysis for (C,D,E) available in supplemental table 3. All panels: n>23; N≥3 biological replicates. * signifies p<0.05, ** signifies p<0.01, *** signifies p<0.001, **** signifies p<0.0001.

Natural condensates are complex, and so it can be difficult to interpret changes in their structure. Therefore, we used a synthetic system to determine how nuclear fluidization by ICP4 impacts condensate formation. Here, a chemical dimerization system initiates multivalent interactions between a fluorescently-tagged coiled-coil protein and nuclear oligomer (Fig. 7A, methods)^64^. This system was previously shown to grow mostly through a nucleation and coalescence mechanism^64^, similar to vRCs^62^.

We transfected HeLa cells with plasmids encoding inducible ICP4 and the two components of the synthetic condensate. We initiated condensate formation by addition of the chemical dimerizer, either after 8h ICP4 induction to drive nuclear fluidization or in control cells, and observed condensate formation kinetics for 1 h (Fig. 7B). We blotted for the components in each condition and determined there was no difference in condensate component production during ICP4 induction compared to uninduced cells (Supplementary Fig. 7A). There was no difference in the average number of condensates per cell in each condition (Fig. 7C), indicating that there was no significant difference in nucleation between the two conditions. However, we observed an increase in condensate growth during ICP4 induction compared to uninduced cells, as measured by average condensate radius (Fig. 7D) and by average condensate intensity (Fig. 7E), where the total intensity for each individual condensate was measured and normalized to the mean intensity of the diffuse phase of the condensate protein. These results indicate that fluidization of the nucleus by ICP4 induction is sufficient to facilitate condensate growth.

## DISCUSSION

The mechanisms that define the physical parameters of the nucleus are poorly understood, perhaps because major perturbations would be highly deleterious. We reasoned that nuclear viruses might encode activities that acutely perturb these properties and thereby provide a path to understand the control of this crucial parameter in normal biology.

We discovered a dramatic increase in mesoscale diffusivity in the nucleus during HSV-1 infection that is linked to an increase in chromatin dynamics, independent of previously described architectural changes. We found that a single factor, the immediate early transcription factor ICP4, can drive this fluidization of the nucleus. We further demonstrated the importance of this fluidization for condensate growth, which has consequences for the maturation of viral replication compartments (vRCs) and thus HSV-1 life cycle progression.

HSV-1 infection was known to have global effects on nuclear size and chromatin structure: host chromatin is marginated leading to enlargement of the interchromatin space^20^, peripheral chromatin restructuring occurs late in infection to allow for virus egress^33,42^, and nuclear volume is increased^20^. However, these changes are all dependent on virus DNA replication^29^, while the increased mesoscale fluidity that we discovered is independent of these processes, indicating a distinct mechanism. Furthermore, nuclear fluidization from a DNA-replication deficient HSV-1 virus or exogenous ICP4 expression both occur without margination of host chromatin or increased nuclear volume.

Although we have yet to elucidate the detailed mechanism by which HSV-1 increases nuclear fluidity, there are several promising possibilities. Chromatin creates significant elastic confinement that reduces the motion of mesoscale particles^65^ and frustrates the growth of mesoscale condensates^15^. Reducing this elastic confinement is one way to increase fluidity. Reduced chromatin confinement can be achieved through an increase in non-thermal motion, for example from increased transcriptional changes or increased chromatin remodeling^13,66–68^. ICP4 binds promiscuously to host chromatin early in infection, and recruits both the transcriptional machinery and chromatin remodelers including the ATPase subunits of the SWI/SNF, INO80, and NuRD complexes^56,69^. We did not detect changes in transcription during ICP4 induction, but we did observe increased motion of bound Halotag-H2A consistent with increased chromatin motion. Recent studies suggest that the SWI/SNF complex decreases the global stiffness of the nucleus^67^, and the NuRD complex increases chromatin motion^68^. These observations are predicted to correlate with increased mesoscale fluidity. Taken together, these observations are consistent with a model where ICP4 fluidizes the nucleus through increased chromatin dynamics.

While existing studies on chromatin remodeling complexes provide a promising pathway for future exploration, the specific effects of ICP4 on SWI/SNF, INO80, and NuRD remain unclear. It is possible that ICP4 manipulates the action of multiple remodeling complexes concurrently. This possibility of engagement with multiple complexes provides interesting possibilities for future work.

HSV-1 might alter the biophysical properties of the nucleus to inhibit host functions or enable viral processes. We speculate that the low mesoscale fluidity in nuclei could be an intrinsic barrier to the replication of all nuclear viruses. This could be particularly relevant in the nuclei of fibroblasts and other epithelial cells resident within dense tissues, where lytic HSV-1 replication typically occurs. Consistent with this idea, viral replication compartment formation and virus production was inefficient at high-density cell cultures that had low mesoscale fluidity. We speculate that ICP4 helps overcome this fundamental physical barrier to virus replication.

Condensate formation is highly sensitive to the physical properties of the cell interior^12,13^. In particular, chromatin has been shown to mechanically frustrate growth of large condensates in the nucleus^15^. Using a synthetic system, we confirmed that ICP4 expression is sufficient to increase the efficiency of formation of large nuclear condensates. Preventing nuclear fluidization also frustrates HSV-1 vRC maturation. This data suggests that HSV-1 fluidizes the nucleus to enable the growth and efficient function of viral replication compartments, thus driving progression of the virus life cycle.

Very little is known about the control of mesoscale nuclear physical properties and their effects on biology. Part of the reason is probably that it is difficult to perturb these properties without loss of viability. This issue can be surmounted by studying lytic viruses, an approach that has led to the discovery of fundamental mechanisms of cellular control. For example, studying sarcoma viruses led to the discovery of the Ras^70,71^ and Src oncogenes^72–74^ that control initiation of cell division, while study of adenovirus led to the discovery of p53 that controls apoptosis^75–78^. We have determined that a single HSV-1 protein, ICP4, increases nuclear fluidity. This provides a path to discover mechanisms by which the physical properties of the nucleus are controlled, and to understand how these properties influence both cellular and viral function.

## METHOD DETAILS

### Cell Culture.

Normal human dermal fibroblasts (Lonza; CC-2509), HeLa (Jef Boeke Lab donation), and HEK293T cells (Jef Boeke Lab donation) were cultured in Dulbecco’s modification of Eagle’s medium (DMEM) (Corning; 10–013-CV) supplemented with 10% (v/v) fetal bovine serum (FBS) (Gemini bio-products; 100–106) and 100 U/mL penicillin-100 μg/mL streptomycin (Corning; MT-30–002-Cl). U2OS cells (ATCC; HTB-96), Vero cells (ATCC; CCL-81), V22 cells, V529 cells, and E5 cells were cultured in DMEM supplemented with 5% (v/v) FBS and 100 U/mL penicillin-100 μg/mL streptomycin. V27 cells were cultured in DMEM supplemented with 5% (v/v) fetal bovine serum FBS and 100 U/mL penicillin-100 μg/mL streptomycin under selection with 400 μg/mL of G418 (Sigma-Aldrich; 04727878001). All cells were grown in a humidified incubator atmosphere at 37°C and 5% CO2.

### Virus growth and infection.

Wild-type HSV-1 KOS was grown on Vero cells. ΔICP4 HSV-1 was grown on E5 cells, both donated by Neal DeLuca (University of Pittsburgh). ΔICP0 HSV-1 was grown on U2OS cells. ΔICP8 HSV-1 was grown on V529 cells, both provided by David Knipe (Harvard Medical School). ΔICP22 HSV-1 was grown on V22 cells; ΔICP27 HSV-1 was grown on V27 cells, all obtained from Steve Rice (University of Minnesota). UV-inactivated virus was prepared by exposing 1 mL layers of virus stock in a six-well dish to six pulses of 0.12 J/cm^2^ of UV light in a Stratalinker (Stratagene). To grow virus stocks, cells were grown to 70–80% confluency in 10 cm dishes, then infected at an MOI of 0.001 in DMEM supplemented with 1% FBS. After 48–72 hours, cells and supernatants were collected and subjected to three freeze-thaw cycles before being aliquoted and stored in −80°C for future use. Virus stock titers were determined using a plaque assay on Vero cells or the appropriate complementing Vero-derived cell line. For all infection experiments, cells were seeded on a 24-well (Cellvis; P24–1.5H-N) or 12-well (Cellvis; P12–1.5H-N) glass bottom plate 24 hours prior to experiment start. Cells were infected at MOI=5 in DMEM with 1% FBS for all virus strains except ΔICP0, which was used at MOI = 10. Media was replaced after 1.5 h of incubation to full media (DMEM + 10% FBS + 1% P/S). Cells were imaged, total protein was harvested for immunoblotting, total RNA was harvested for RNA extraction, or cells were fixed for immunofluorescence at indicated timepoints. For experiments under confluent conditions, cells were seeded at 100% confluency 48 h prior to the start of the experiment. Cells were infected with MOI = 5. MOI was calculated based on hemocytometer counts of wells at 70–80% confluence and 100% confluence, respectively.

### Lentivirus/Retrovirus creation.

HEK293T cells (9 × 10^6^ per 15 cm dish) were plated in antibiotic free DMEM (Gibco, Cat. No. 11995073) supplemented with 10% FBS. The next day, cells were transfected with appropriate transgene plasmid together with packaging plasmids (psPAX2 and pMD2.G for lentivirus, VSV-G and GP for retrovirus), using fuGENE HD transfection reagent (Promega, Cat. no. E2312) following manufacturer’s protocol. 24 h later, 15 mL antibiotic free DMEM was replaced, and the supernatants were collected at 72 h post-transfection, aliquoted and stored at −80°C until later use. Both lentivirus and retrovirus were introduced into NHDF wild type and NHDF-nucPfV cell lines of interest via reverse transduction with 5 mL of virus in fresh media in the presence of 10 μg/mL polybrene (Sigma-Aldrich; TR-1003-G), replacing media after 24 h. Cells were selected with puromycin for three days. 24 h prior to experiment start, cells were split to a 24-well (Cellvis; P24–1.5H-N) or 12-well (Cellvis; P12–1.5H-N) glass bottom plate. Cells were freshly transduced with lentivirus for each new experiment and used within 2 passages of transduction. For cells transduced with retrovirus, after selecting antibiotic resistant cell populations, they were frozen in 10% DMSO (Sigma-Aldrich, Cat. no. D2650– 100) in FBS and thawed for use in experiments whenever needed.

### Plasmid construction.

Sequence blocks encoding codon optimized HSV-1 ICP4 and ICP8 (based on KOS strain) were obtained from Integrated DNA technologies. The custom vector for a Tet-inducible ICP4 expression plasmid was from VectorBuilder (VB211009–1101fac: https://en.vectorbuilder.com/). A codon-optimized ICP8 custom gene block was purchased from Twist Bioscience (https://ecommerce.twistdna.com/). To construct lentiviral vectors expressing ICP4 and ICP8, respectively, the assembled coding sequences were incorporated into a Tet-inducible lentivirus vector via Gibson assembly.

To generate ICP4 mutant proteins, primers were generated to amplify segments lacking either the C-terminal activation domain or the first 90 amino acids of the N-terminal activation domain of ICP4 from the codon optimized ICP4 present in pLH2488^79,80^. pLH2631 and pLH2632 were made using Gibson assembly of the PCR DNA fragments encoding the KOZAK sequence and ICP4-ΔNTA or ICP4-ΔCTA respectively. The pLVX-TetOne-puro plasmid backbone was digested from pLH2537 using EcoRI and BamHI, then purified and ligated to the ICP4-ΔNTA and ICP4-ΔCTA fragments. Localized mutations in the DNA-binding domain of ICP4 (ICP4-*m*DBD-RR/LL and ICP4-*m*DBD-KG/NC) were introduced into pLH2488 using overlapping primers containing the mutation of interest to generate two PCR products that had homology to one another^48^. The products were then assembled using NEB HiFi DNA assembly to generate pLH2658 and pLH2659. All constructs were confirmed by DNA sequencing.

### Transient transfection.

For nucGEM experiments in HeLa cells, cells were seeded at 60%–70% confluency in a 6-well tissue culture plate (CELLTREAT; 229106) and transfected the next day with 1 μg of plasmid DNA (pLH2112) per well using fuGENE HD reagent per manufacturer guidelines. For artificial condensate experiments, 6 × 10^5^ HeLa cells were reverse transfected in a 6-well tissue culture plate (CELLTREAT; 229106) with 0.8 μg of ICP4 expressing plasmid (pLH2112) and 0.5 μg of each plasmid encoding an artificial condensate component (pLH2712 and pLH2714). pLH2712 contains the nuclear hexamer HO Tag3 fused to HaloTag. pLH2714 contains the phase-separating coiled-coil protein Mad1 with an N-terminal fusion to mScarlet-eDHFR^81^ to facilitate visualization and binding to the HaloTag of pLH2712 upon chemical dimerization. Culture media was replaced after 12 h and cells were split into a 24-well (Cellvis; P24–1.5H-N) or 12-well (Cellvis; P12–1.5H-N) glass bottom plate for imaging 24–48 h post-transfection. Imaging experiments were usually carried out between 48 and 72 h post-transfection.

### Induction and chemical treatments.

For all induction experiments, cells were seeded on a 24-well (Cellvis; P24–1.5H-N) or 12-well (Cellvis; P12–1.5H-N) glass bottom plate 24 hours prior to experiment start as previously described. For experiments blocking virus DNA replication, cells were treated with 300 μg/mL phosphonoacetic acid (PAA) (Sigma-Aldrich; 284270) beginning with virus addition. PAA was refreshed when virus inoculum was removed. To induce hyperosmotic stress, 150 mM sorbitol was added at 2.5 hpi, which roughly correlates with the onset of virus E protein expression as determined by immunoblotting. For doxycycline (dox) induction of ectopically expressed proteins, cells were treated with 3 μg/mL dox for 9 h prior to imaging or further processing. To perform single particle tracking of HaloTag-H2A, HaloTag-H2A was labeled with 10 pM JFX646 (gift of Janelia HHMI/Lavis Lab) in cell culture media for 30 minutes, then washed three times with PBS (Corning; 21–040-CV) before replacing with fresh media and proceeding to infection or induction as indicated.

### Microscopy.

For nucGEM experiments, artificial condensate assay experiments, as well as immunofluorescence imaging experiments, cells were mounted on a Nikon Ti2 X1 spinning disc confocal microscope and images were captured using a Prime 95B scMOS camera (Photometrics) with a 60x/1.49 numerical aperture objective lens with DIC capability. Fluorescence signals were obtained using 405 nm, 488 nm, 561 nm, and 640 nm lasers with the following emission filters: 455/50, 525/36, 605/52, and 705/72 (Chroma Technology Corp). For live cell imaging, the microscope was equipped with an incubator to maintain 37°C and 5% CO_2_. Cells were stained with a nuclear stain (SiR-DNA (Cytoskeleton; CY-SC007) or Hoechst (Thermo Fisher Scientific; 62249)) if applicable, then imaged at indicated timepoints. The nucGEMs were imaged at a rate of one image every 10 ms.

For single particle tracking of Halo-H2A, cells were mounted on a Nikon Ti Eclipse microscope equipped with a motorized Ti H-TIRF module and an Andor Zyla sCMOS camera behind a 0.7x relay magnification lens. Movies were acquired in highly inclined laminated optical sheet (HILO) mode as described in Tokunaga et al. ^82^. Data was collected using a CFI Apo 60× 1.49 NA TIRF objective and a quad-band STORM ultra-high S/N dichroic/emission filter (Nikon, 97335). Excitation was provided by a 640 nm laser (LUNF-XL, Nikon), and total internal reflection was calibrated using Nikon Elements software (insert version number). For HILO acquisition, the incident angle was manually adjusted to optimize H2A-HaloTag signal-to-noise, typically 5–10° below the critical angle and a 512×512 region of interest (ROI) was selected around the most evenly illuminated field of view. For our 250 ms timescale, we collected images at 25 ms exposure for 2,000 frames (40 Hz). To analyze data at 4 Hz (2500 ms timescale), max projections were created of every 10 slices to form a new collection of 200 total frames.

For live imaging of artificial condensate formation, the cells were mounted on a Nikon spinning disk confocal scanning microscope as previously described. Cells without condensates, but with high expression as determined by gating on the look up table (LUT) of both the anchor and the phase separating protein were selected and image acquisition began. At the end of the first time loop, an additional volume of growth media containing the chemical dimerizer TMP-Fluorobenzamide-Halo (TFH) to a final concentration of 50 μM was added to the stage to induce condensate formation in the mounted cells^81^. Z-stack images were collected with 1 μm spacing for a total depth of up to 12 μm, at 2 minute intervals for 1 hour or at 4 minute intervals for 4 hours.

### Particle tracking.

All trajectories from nucGEMs and Halo-H2A single particle tracking were analyzed with the GEMspa (GEM single particle analysis) software package that we are developing in house (https://github.com/liamholtlab/GEMspa, ^83^). Mean-square displacement (MSD) was calculated for every 2D trajectory, and trajectories continuously followed for more than 10 time points were used to fit with linear time dependence based on the first 10 time intervals to quantify time-averaged MSD: MSD(T) = 4D_eff_T, where T is the imaging time interval and D_eff_ is the effective diffusivity with the unit of μm^2^/s. To determine the ensemble-time-averaged mean-square displacement (MSD), all trajectories were fitted with MSD(τ)_T−ens_=4Dτα where α is the anomalous exponent, with α = 1 being Brownian motion, α < 1 suggests sub-diffusive motion and α > 1 as super-diffusive motion. To generate region of interest (ROI) for marking individual cells, cellpose python package (https://github.com/mouseland/cellpose, ^84^) was used to segment based on the nuclear stain or the average projection intensity of Halo-H2A particles. These ROI were then input into GEMspa to quantify single cell effective diffusion, with at least three trajectories averaged for individual yeast cells. We used the median value of D_eff_ for single cell data to represent each condition.

### Condensate analysis.

For analysis of live timelapses of artificial condensates, maximum projections of the z-stack images were pre-processed using FIJI. Condensates were identified and their parameters tracked using TrackMate plug-in in FIJI^85^. Outputs from TrackMate were filtered and analyzed using a jupyter notebook in VS Code. For immunofluorescence imaging of fixed condensates, z-stack images were collected with 0.5 μm spacing for a total depth of 20 μm. To generate region of interest (ROI) for marking individual cells, cellpose python package (https://github.com/mouseland/cellpose, ^84^) was used to segment based on the nuclear stain. The resulting images were analyzed with foci-counting, a software package that we are developing in house: https://github.com/liamholtlab/foci_counting.

### Immunofluorescence.

Cells were treated as indicated, then immediately fixed at indicated timepoints with 4% paraformaldehyde (Electron Microscopy Sciences, Cat. No. 15714) for 10 min at room temperature. The cells were subsequently washed three times with 1x PBS, and permeabilized with 0.5% Triton X-100 (Fisher Scientific, Cat. No. 9002–93-1) in 1x PBS for 15 min at room temperature. After blocking with 4% FBS in PBS for 1 h, primary antibodies (1:500 dilution) were applied overnight at 4°C. The next day, the cells were washed three times with 1x PBS and incubated with secondary antibodies (1:500 dilution) in 4% FBS in PBS at room temperature for 1 h in the dark. After washing three times with 1x PBS, cells were stained with SiR-DNA (1:5000 dilution) or 1 μM Hoechst 33342 (Thermo Fisher Scientific 62249). Cells were subsequently stored in 1x PBS at 4°C in the dark until imaging. The fixed plates of cells were mounted on a Nikon spinning disk confocal scanning microscope. Fluorescence signals were obtained using 405 nm, 488 nm, 561 nm, and 640 nm lasers with the following emission filters: 455/50, 525/36, 605/52, and 705/72 (Chroma Technology Corp). Images were captured using a Prime 95B scMOS camera (Photometrics) with a 60x/1.49 numerical aperture objective lens with DIC capability. To calculate cell volume, vRC volume, vRC mean intensity, condensate count, condensate mean intensity, and background intensity, 20-μm Z-stacks were taken with 0.5-μm steps between frames, and images were analyzed with foci-counting.

### Immunoblotting.

Total cellular protein was collected by lysis in sample buffer (62.5 mM Tris-HCl pH 6.8, 2% SDS, 10% glycerol, 0.7M β-mercaptoethanol) followed by boiling for 5 minutes. Lysates were fractionated by sodium dodecyl sulfate polyacrylamide gel electrophoresis (SDS-PAGE) and transferred to nitrocellulose membranes. Membranes were blocked in 5% non-fat milk in TBST for 1 h at room temperature and incubated in primary antibody overnight at 4°C. Primary antibodies were detected using either anti-rabbit IgG HRP or anti-mouse IgG HRP secondary antibodies and visualized by chemiluminescent detection using an Invitrogen iBright FL1000 Imaging System.

### Quantification and stats.

Images were quantitated using FIJI plugin MOSAIC for ImageJ^86^. Single particle tracking data was analyzed with GEMspa^83^. Nuclear volumes and vRC volumes were quantified using foci-counting, a software package developed in-house by Sarah Keegan (https://github.com/liamholtlab/foci_counting). Artificial condensates were analyzed and quantified with TrackMate Fiji plug-in^85^. Outputs from TrackMate were filtered and analyzed using a jupyter notebook in VS Code. ChatGPT (GPT-4-turbo, OpenAI) was occasionally used for code debugging and optimization. Graphs were generated by GraphPad Prism 10 (GraphPad Software). Statistical analysis was performed with GraphPad Prism 10 (GraphPad Software). We used the Kruskal-Wallis test for all one-way comparisons. All two-way ANOVAs were followed by Tukey’s post hoc test to correct for multiple comparisons unless otherwise indicated. Further statistical details can be found in the figure legends.

## Supplementary Material

Supplement 1

## Figures and Tables

**Resource table:** 

REAGENT	SOURCE	IDENTIFIER
**Antibodies**		
ICP4	abcam	ab6514
ICP8	abcam	ab20193
GAPDH	Cell Signaling	2118S
gC	abcam	ab6509
UL42	Santa Cruz	SC-53331
VP16	abcam	ab110226
ICP0	abcam	ab6513
Halo	Promega	G9211
RFP/mScarlet	Rockland	600-401-379
ICP27	abcam	ab31631
**Plasmids & Recombinant DNA**		
pMD2.G	Addgene #12259	pLH1863
psPAX2	Addgene #12260	pLH1864
TetOn-HSV1-ICP4-BFP2	Vector Builder Custom order: VB211009-1101fac	pLH2112
pUBC-NLS-PfV-TSapphire	Liam Holt (NYU Langone Health)	pLH2169
pLVX-TetOne-puro-ICP4	Liam Holt (NYU Langone Health)	pLH2488
pVSV-G	Addgene #138479	pLH2496
gag/pol	Addgene #14887	pLH2497
pBABE-puro-Halo-H2A	Eli Rothenberg (NYU Langone Health)	pLH2498
pLVX-TetOne-puro	Meike Dittman (NYU Langone Health)	pLH2537
pLVX-TetOne-puro-ICP4-ΔNTA	Liam Holt (NYU Langone Health)	pLH2631
pLVX-TetOne-puro-ICP4-ΔCTA	Liam Holt (NYU Langone Health)	pLH2632
pLVX-TetOne-puro-ICP4-mDBD-RR/LL	Liam Holt (NYU Langone Health)	pLH2658
pLVX-TetOne-puro-ICP4-mDBD-KG/NC	Liam Holt (NYU Langone Health)	pLH2659
p503 jt74 RMCE miRFP670-Halo-HOTag3	Huaiying Zhang (Carnegie Mellon University)	pLH2712
p403 jt61 RMCE mScarlet-eDHFR-Mad1	Huaiying Zhang (Carnegie Mellon University)	pLH2714
pLVX-TetOne-puro-ICP8	Liam Holt (NYU Langone Health)	pLH2733
**Bacterial and Virus Strains**		
KOS	Priscilla Schaffer (University of Pennsylvania)	HSV-1 Wild Type
7134	Priscilla Schaffer (University of Pennsylvania)	HSV-1 ΔICP0
N12	Neal DeLuca (University of Pittsburgh)	HSV-1 ΔICP4
d22lacZ #8	Steve Rice (University of Minnesota)	HSV-1 ΔICP22
d27-1	Steve Rice (University of Minnesota)	HSV-1 ΔICP27
d301	David Knipe (Harvard Medical School)	HSV-1 ΔICP8
n6	Neal DeLuca (University of Pittsburgh)	HSV-1 ΔNTA
n208	Neal DeLuca (University of Pittsburgh)	HSV-1 ΔCTA
Rosetta (DE3) Competent Cells - Novagen	Millipore Sigma	70954-3
**Chemicals, Peptides, and Recombinant Proteins**		
Cycloheximide	Sigma	C104450
Phosphonoacetic acid, 98%	Sigma-Aldrich	284270-10G
Doxycycline	Sigma-Aldrich	D5207-5G
D-Sorbitol	Sigma-Aldrich	S1876
JFX 650 HaloTag Ligand	Janelia/HHMI/Lavis Lab	
TMP-Fluorobenzamide-Halo (TFH)	Huaiying Zhang (Carnegie Mellon University)	
Tri*PPP*ro 2TCOa-dCTP	UHH, AK Meier	
HD653 far red dye	UHH, AK Meier	IK341
Puromycin	MedChem Express	HY-B1743A
Polybrene Transfection Reagent	Sigma-Aldrich	TR-1003-G
SiR-DNA	Cytoskeleton	CY-SC007
DAPI	Fisher Scientific	D1306
Hoechst 33342	Thermo Scientific	62249
G-418 Solution	Sigma-Aldrich	04727878001
Fugene	Promega	E2312
**Critical Commercial Assays**		
Gibson Assembly Master Mix	NEB	E2611S
NEBuilder HiFi DNA Assembly Master Mix	NEB	E2621S
Q5 DNA Polymerase	NEB	M0491S
Phusion DNA Polymerase	Thermo Scientific	F530S
**Experimental Models: Cell Lines**		
Normal human dermal fibroblasts (NHDF)	Lonza	CC-2509
NHDF-nucPfV (pLH2169 lentivirus)	Liam Holt (NYU Langone Health)	N/A
NHDF-Halo-H2A (pLH2498 lentivirus)	Liam Holt (NYU Langone Health)	N/A
NHDF-nucPfV-ICP4 (pLH2169 & pLH2488 lentivirus)	Liam Holt (NYU Langone Health)	N/A
NHDF-nucPfV-ICP8 (pLH2169 & pLH2733 lentivirus)	Liam Holt (NYU Langone Health)	N/A
NHDF-nucPfV-ICP4-ΔNTA (pLH2169 & pLH2631 lentivirus)	Liam Holt (NYU Langone Health)	N/A
NHDF-nucPfV-ICP4-ΔCTA (PLH2169 & pLH2632 lentivirus)	Liam Holt (NYU Langone Health)	N/A
NHDF-nucPfV-ICP4-mDBD-RR/LL (pLH2169 & pLH2658 lentivirus)	Liam Holt (NYU Langone Health)	N/A
NHDF-nucPfV-ICP4-mDBD-KG/NC (pLH2169 & pLH2659 lentivirus)	Liam Holt (NYU Langone Health)	N/A
NHDF-Halo-H2A-ICP4 (pLH2498 & pLH2488 lentivirus)	Liam Holt (NYU Langone Health)	N/A
NHDF-Halo-H2A-ICP4-ΔNTA (pLH2498 & pLH2631 lentivirus)	Liam Holt (NYU Langone Health)	N/A
HeLa	Jef Boeke (NYU Langone Health)	N/A
HeLa-nucPfV (pLH2169 lentivirus)	Liam Holt (NYU Langone Health)	N/A
HeLa-nucPfV-ICP4 (pLH2169 lentivirus & pLH21l2 transient transfection)	Liam Holt (NYU Langone Health)	N/A
HeLa-ICP4-Mad1-HOTag-miRF P670 (pLH2112, pLH2712, pLH2714 transient transfection)	Liam Holt (NYU Langone Health)	N/A
HeLa-ICP4-Mad1-HOTag-eGFP (pLH2112, pLH2713, pLH2714 transient transfection)	Liam Holt (NYU Langone Health)	N/A
HEK293T	Jef Boeke (NYU Langone Health)	N/A
E5	Neal DeLuca (University of Pittsburgh)	N/A
African green monkey cells (Vero)	ATCC	CCL-81
V22	Steve Rice (University of Minnesota)	N/A
V27	Steve Rice (University of Minnesota)	N/A
V529	David Knipe (Harvard School of Medicine)	N/A
**Software and Algorithms**		
Python	Open source	https://www.python.org/downloads/release/python-3107/
FIJI (FIJI is just ImageJ)	^87^	https://imagej.net/software/fiji/
MOSAIC for ImageJ	^86^	https://github.com/mosaic-group/MosaicSuite
foci-counting		https://github.com/liamholtlab/foci_counting
GEMSpa	^83^	https://github.com/liamholtlab/napari-gemspa (napari plugin), https://github.com/liamholtlab/GEMspa (stand alone)
Cellpose	^84^	https://cellpose.readthedocs.io/en/latest/#
TrackMate	^85^	https://github.com/trackmate-sc/TrackMate
Nikon Elements	Nikon Instruments (2017)	https://www.nikoninstruments.com/Products/Software

## References

[1] Zhou H.-X., Rivas G., and Minton A.P. (2008). Macromolecular crowding and confinement: biochemical, biophysical, and potential physiological consequences. Annu. Rev. Biophys. 37, 375–397. 10.1146/annurev.biophys.37.032807.125817. 18573087 PMC2826134

[2] Rivas G., and Minton A.P. (2016). Macromolecular Crowding In Vitro, In Vivo, and In Between. Trends Biochem. Sci. 41, 970–981. 10.1016/j.tibs.2016.08.013. 27669651 PMC5804487

[3] Mackintosh F.C., and Schmidt C.F. (2010). Active cellular materials. Curr. Opin. Cell Biol. 22, 29–35. 10.1016/j.ceb.2010.01.002. 20089390

[4] Luby-Phelps K., Taylor D.L., and Lanni F. (1986). Probing the structure of cytoplasm. J. Cell Biol. 102, 2015–2022. 2423529 10.1083/jcb.102.6.2015 PMC2114258

[5] Bonucci M., Shu T., and Holt L.J. (2023). How it feels in a cell. Trends Cell Biol. 33, 924–938. 10.1016/j.tcb.2023.05.002. 37286396 PMC10592589

[6] Parry B.R., Surovtsev I.V., Cabeen M.T., O’Hern C.S., Dufresne E.R., and Jacobs-Wagner C. (2014). The bacterial cytoplasm has glass-like properties and is fluidized by metabolic activity. Cell 156, 183–194. 24361104 10.1016/j.cell.2013.11.028 PMC3956598

[7] Nishizawa K., Fujiwara K., Ikenaga M., Nakajo N., Yanagisawa M., and Mizuno D. (2017). Universal glass-forming behavior of in vitro and living cytoplasm. Sci. Rep. 7, 15143. 10.1038/s41598-017-14883-y. 29123156 PMC5680342

[8] Alric B., Formosa-Dague C., Dague E., Holt L.J., and Delarue M. (2022). Macromolecular crowding limits growth under pressure. Nat. Phys. 18, 411–416. 10.1038/s41567-022-01506-1. 37152719 PMC10162713

[9] Neurohr G.E., Terry R.L., Brittingham G.P., Lengefeld J., Holt L.J., and Amon A. (2019). Excessive Cell Growth Causes Cytoplasm Dilution And Contributes to Senescence . Cell 5, 1083–1097. 10.1016/j.cell.2019.01.018. PMC6386581 30739799

[10] Boeynaems S., Chong S., Gsponer J., Holt L., Milovanovic D., Mitrea D.M., Mueller-Cajar O., Portz B., Reilly J.F., Reinkemeier C.D., (2023). Phase Separation in Biology and Disease; Current Perspectives and Open Questions. J. Mol. Biol. 435, 167971. 10.1016/j.jmb.2023.167971. 36690068 PMC9970028

[11] Delarue M., Brittingham G.P., Pfeffer S., Surovtsev I.V., Pinglay S., Kennedy K.J., Schaffer M., Gutierrez J.I., Sang D., Poterewicz G., (2018). mTORC1 Controls Phase Separation and the Biophysical Properties of the Cytoplasm by Tuning Crowding. Cell 174, 338–349.e20. 29937223 10.1016/j.cell.2018.05.042 PMC10080728

[12] Shu T., Mitra G., Alberts J., Viana M.P., Levy E.D., Hocky G.M., and Holt L.J. (2024). Mesoscale molecular assembly is favored by the active, crowded cytoplasm. PRX Life 2. 10.1103/prxlife.2.033001. PMC11952695 40162127

[13] Xie Y., Shu T., Liu T., Spindler M.-C., Mahamid J., Hocky G.M., Gresham D., and Holt L.J. (2024). Polysome collapse and RNA condensation fluidize the cytoplasm. Mol. Cell 84, 2698–2716.e9. 10.1016/j.molcel.2024.06.024. 39059370 PMC11539954

[14] Banerjee D.S., Chigumira T., Lackner R.M., Kratz J.C., Chenoweth D.M., Banerjee S., and Zhang H. (2024). Interplay of condensate material properties and chromatin heterogeneity governs nuclear condensate ripening. bioRxivorg. 10.1101/2024.05.07.593010.

[15] Zhang Y., Lee D.S.W., Meir Y., Brangwynne C.P., and Wingreen N.S. (2021). Mechanical Frustration of Phase Separation in the Cell Nucleus by Chromatin. Phys. Rev. Lett. 126, 258102. 10.1103/PhysRevLett.126.258102. 34241518 PMC8604804

[16] Abrisch R.G., Eidem T.M., Yakovchuk P., Kugel J.F., and Goodrich J.A. (2015). Infection by herpes simplex virus 1 causes near-complete loss of RNA polymerase II occupancy on the host cell genome. J. Virol. 90, 2503–2513. 10.1128/JVI.02665-15. 26676778 PMC4810688

[17] Friedel C.C., Whisnant A.W., Djakovic L., Rutkowski A.J., Friedl M.-S., Kluge M., Williamson J.C., Sai S., Vidal R.O., Sauer S., (2021). Dissecting herpes simplex virus 1-induced host shutoff at the RNA level. J. Virol. 95. 10.1128/JVI.01399-20. PMC7925104 33148793

[18] Banerjee A.K., Blanco M.R., Bruce E.A., Honson D.D., Chen L.M., Chow A., Bhat P., Ollikainen N., Quinodoz S.A., Loney C., (2020). SARS-CoV-2 disrupts splicing, translation, and protein trafficking to suppress host defenses. Cell 183, 1325–1339.e21. 10.1016/j.cell.2020.10.004. 33080218 PMC7543886

[19] Rosemarie Q., and Sugden B. (2023). Five families of diverse DNA viruses comprehensively restructure the nucleus. PLoS Biol. 21, e3002347. 10.1371/journal.pbio.3002347. 37930945 PMC10627436

[20] Monier K., Armas J.C., Etteldorf S., Ghazal P., and Sullivan K.F. (2000). Annexation of the interchromosomal space during viral infection. Nat. Cell Biol. 2, 661–665. 10.1038/35023615. 10980708

[21] Strang B.L., Boulant S., Chang L., Knipe D.M., Kirchhausen T., and Coen D.M. (2012). Human cytomegalovirus UL44 concentrates at the periphery of replication compartments, the site of viral DNA synthesis. J. Virol. 86, 2089–2095. 10.1128/JVI.06720-11. 22156516 PMC3302373

[22] Herrera F.J., and Triezenberg S.J. (2004). VP16-dependent association of chromatin-modifying coactivators and underrepresentation of histones at immediate-early gene promoters during herpes simplex virus infection. J. Virol. 78, 9689–9696. 10.1128/JVI.78.18.9689-9696.2004. 15331701 PMC515004

[23] Horwitz G.A., Zhang K., McBrian M.A., Grunstein M., Kurdistani S.K., and Berk A.J. (2008). Adenovirus small e1a alters global patterns of histone modification. Science 321, 1084–1085. 10.1126/science.1155544. 18719283 PMC2756290

[24] Castillo-González C., Liu X., Huang C., Zhao C., Ma Z., Hu T., Sun F., Zhou Y., Zhou X., Wang X.-J., (2015). Geminivirus-encoded TrAP suppressor inhibits the histone methyltransferase SUVH4/KYP to counter host defense. Elife 4, e06671. 10.7554/eLife.06671. 26344546 PMC4606454

[25] Nikolic J., Le Bars R., Lama Z., Scrima N., Lagaudrière-Gesbert C., Gaudin Y., and Blondel D. (2017). Negri bodies are viral factories with properties of liquid organelles. Nat. Commun. 8, 58. 10.1038/s41467-017-00102-9. 28680096 PMC5498545

[26] Heinrich B.S., Maliga Z., Stein D.A., Hyman A.A., and Whelan S.P.J. (2018). Phase transitions drive the formation of vesicular stomatitis virus replication compartments. MBio 9. 10.1128/mBio.02290-17. PMC6123442 30181255

[27] Chen H., Cui Y., Han X., Hu W., Sun M., Zhang Y., Wang P.-H., Song G., Chen W., and Lou J. (2020). Liquid-liquid phase separation by SARS-CoV-2 nucleocapsid protein and RNA. Cell Res. 30, 1143–1145. 10.1038/s41422-020-00408-2. 32901111 PMC7477871

[28] Knipe D.M., Ekaterina E. Heldwein, Mohr I.J., and Sodroski C.N. (2022). Herpes Simplex Viruses: Mechanisms of Lytic and Latent Infection. In Field’s Virology, Volume 2: DNA Viruses, Howley Peter M., Knipe David M., Blossom Damania, Cohen Jeffrey, Whelan Sean P. J., Freed Eric O., and Enquist L.W., eds. (Wolters Kluwer), pp. 235–296.

[29] Aho V., Mäntylä E., Ekman A., Hakanen S., Mattola S., Chen J.-H., Weinhardt V., Ruokolainen V., Sodeik B., Larabell C., (2019). Quantitative Microscopy Reveals Stepwise Alteration of Chromatin Structure during Herpesvirus Infection. Viruses 11, 935. 10.3390/v11100935. 31614678 PMC6832731

[30] James C., Harfouche M., Welton N.J., Turner K.M., Abu-Raddad L.J., Gottlieb S.L., and Looker K.J. (2020). Herpes simplex virus: global infection prevalence and incidence estimates, 2016. Bull. World Health Organ. 98, 315–329. 10.2471/BLT.19.237149. 32514197 PMC7265941

[31] Zhu S., and Viejo-Borbolla A. (2021). Pathogenesis and virulence of herpes simplex virus. Virulence 12, 2670–2702. 10.1080/21505594.2021.1982373. 34676800 PMC8923070

[32] Denes C.E., Everett R.D., and Diefenbach R.J. (2020). Tour de herpes: Cycling through the life and biology of HSV-1. Methods Mol. Biol. 2060, 1–30. 10.1007/978-1-4939-9814-2_1. 31617170

[33] Bosse J.B., Hogue I.B., Feric M., Thiberge S.Y., Sodeik B., Brangwynne C.P., and Enquist L.W. (2015). Remodeling nuclear architecture allows efficient transport of herpesvirus capsids by diffusion. Proc. Natl. Acad. Sci. U. S. A. 112, E5725–E5733. 10.1073/pnas.1513876112. 26438852 PMC4620878

[34] Liptak L.M., Uprichard S.L., and Knipe D.M. (1996). Functional order of assembly of herpes simplex virus DNA replication proteins into prereplicative site structures. J. Virol. 70, 1759–1767. 10.1128/JVI.70.3.1759-1767.1996. 8627698 PMC190001

[35] de Bruyn Kops A., and Knipe D.M. (1994). Preexisting nuclear architecture defines the intranuclear location of herpesvirus DNA replication structures. J. Virol. 68, 3512–3526. 8189490 10.1128/jvi.68.6.3512-3526.1994 PMC236855

[36] Charman M., and Weitzman M.D. (2020). Replication Compartments of DNA Viruses in the Nucleus: Location, Location, Location. Viruses 12, 151. 10.3390/v12020151. 32013091 PMC7077188

[37] Shu T., Szórádi T., Kidiyoor G.R., Xie Y., Herzog N.L., Bazley A., Bonucci M., Keegan S., Saxena S., Ettefa F., (2022). nucGEMs probe the biophysical properties of the nucleoplasm. bioRxiv. 10.1101/2021.11.18.469159.

[38] Akita F., Chong K.T., Tanaka H., Yamashita E., Miyazaki N., Nakaishi Y., Suzuki M., Namba K., Ono Y., Tsukihara T., (2007). The crystal structure of a virus-like particle from the hyperthermophilic archaeon Pyrococcus furiosus provides insight into the evolution of viruses. J. Mol. Biol. 368, 1469–1483. 10.1016/j.jmb.2007.02.075. 17397865

[39] Delarue M., Brittingham G.P., Pfeffer S., Surovtsev I.V., Pinglay S., Kennedy K.J., Schaffer M., Gutierrez J.I., Sang D., Poterewicz G., (2018). mTORC1 controls phase separation and the biophysical properties of the cytoplasm by tuning crowding. Cell 174, 338–349.e20. 10.1016/j.cell.2018.05.042. 29937223 PMC10080728

[40] Shen H., Tauzin L.J., Baiyasi R., Wang W., Moringo N., Shuang B., and Landes C.F. (2017). Single particle tracking: From theory to biophysical applications. Chem. Rev. 117, 7331–7376. 10.1021/acs.chemrev.6b00815. 28520419

[41] Requena B., Masó-Orriols S., Bertran J., Lewenstein M., Manzo C., and Muñoz-Gil G. (2023). Inferring pointwise diffusion properties of single trajectories with deep learning. Biophys. J. 122, 4360–4369. 10.1016/j.bpj.2023.10.015. 37853693 PMC10698275

[42] Lewis H.C., Kelnhofer-Millevolte L.E., Brinkley M.R., Arbach H.E., Arnold E.A., Sanders S., Bosse J.B., Ramachandran S., and Avgousti D.C. (2023). HSV-1 exploits host heterochromatin for nuclear egress. J. Cell Biol. 222, e202304106. 10.1083/jcb.202304106. 37516914 PMC10373338

[43] Nguyen L.H., Knipe D.M., and Finberg R.W. (1992). Replication-defective mutants of herpes simplex virus (HSV) induce cellular immunity and protect against lethal HSV infection. J. Virol. 66, 7067–7072. 10.1128/JVI.66.12.7067-7072.1992. 1331509 PMC240374

[44] Hirai K. (1979). The physical state of herpes simplex virus DNA in infected human cells. Microbiol. Immunol. 23, 749–761. 10.1111/j.1348-0421.1979.tb00517.x. 232235

[45] Simpson-Holley M., Colgrove R.C., Nalepa G., Harper J.W., and Knipe D.M. (2005). Identification and functional evaluation of cellular and viral factors involved in the alteration of nuclear architecture during herpes simplex virus 1 infection. J. Virol. 79, 12840–12851. 10.1128/JVI.79.20.12840-12851.2005. 16188986 PMC1235858

[46] Tunnicliffe R.B., Lockhart-Cairns M.P., Levy C., Mould A.P., Jowitt T.A., Sito H., Baldock C., Sandri-Goldin R.M., and Golovanov A.P. (2017). The herpes viral transcription factor ICP4 forms a novel DNA recognition complex. Nucleic Acids Res. 45, 8064–8078. 10.1093/nar/gkx419. 28505309 PMC5737704

[47] DeLuca N.A., and Schaffer P.A. (1988). Physical and functional domains of the herpes simplex virus transcriptional regulatory protein ICP4. J. Virol. 62, 732–743. 10.1128/JVI.62.3.732-743.1988. 2828668 PMC253626

[48] Allen K.E., and Everett R.D. (1997). Mutations which alter the DNA binding properties of the herpes simplex virus type 1 transactivating protein Vmw175 also affect its ability to support virus replication. J. Gen. Virol. 78, 2913–2922. 10.1099/0022-1317-78-11-2913. 9367379

[49] Kuddus R.H., and DeLuca N.A. (2007). DNA-dependent oligomerization of herpes simplex virus type 1 regulatory protein ICP4. J. Virol. 81, 9230–9237. 10.1128/JVI.01054-07. 17581987 PMC1951460

[50] Dremel S.E., and DeLuca N.A. (2019). Herpes simplex viral nucleoprotein creates a competitive transcriptional environment facilitating robust viral transcription and host shut off. Elife 8, e51109. 10.7554/eLife.51109. 31638576 PMC6805162

[51] Jao C.Y., and Salic A. (2008). Exploring RNA transcription and turnover in vivo by using click chemistry. Proc. Natl. Acad. Sci. U. S. A. 105, 15779–15784. 10.1073/pnas.0808480105. 18840688 PMC2572917

[52] Breinbauer R., and Köhn M. (2003). Azide-alkyne coupling: a powerful reaction for bioconjugate chemistry. Chembiochem 4, 1147–1149. 10.1002/cbic.200300705. 14613105

[53] Moore C., Wong E., Kaur U., Chio U.S., Zhou Z., Ostrowski M., Wu K., Irkliyenko I., Wang S., Ramani V., (2024). ATP-dependent remodeling of chromatin condensates uncovers distinct mesoscale effects of two remodelers. bioRxivorg. 10.1101/2024.09.10.611504. PMC13027391 41037607

[54] Zhao J.Z., Xia J., and Brangwynne C.P. (2024). Chromatin compaction during confined cell migration induces and reshapes nuclear condensates. Nat. Commun. 15, 9964. 10.1038/s41467-024-54120-5. 39557835 PMC11574006

[55] Albert B., Léger-Silvestre I., Normand C., and Gadal O. (2012). Nuclear organization and chromatin dynamics in yeast: biophysical models or biologically driven interactions? Biochim. Biophys. Acta 1819, 468–481. 10.1016/j.bbagrm.2011.12.010. 22245105

[56] Wagner L.M., and DeLuca N.A. (2013). Temporal Association of Herpes Simplex Virus ICP4 with Cellular Complexes Functioning at Multiple Steps in PolII Transcription. PLoS One 8, e78242. 10.1371/journal.pone.0078242. 24147125 PMC3795685

[57] Los G.V., Encell L.P., McDougall M.G., Hartzell D.D., Karassina N., Zimprich C., Wood M.G., Learish R., Ohana R.F., Urh M., (2008). HaloTag: a novel protein labeling technology for cell imaging and protein analysis. ACS Chem. Biol. 3, 373–382. 10.1021/cb800025k. 18533659

[58] Devany J., Falk M.J., Holt L.J., Murugan A., and Gardel M.L. (2023). Epithelial tissue confinement inhibits cell growth and leads to volume-reducing divisions. Dev. Cell 58, 1462–1476.e8. 10.1016/j.devcel.2023.05.018. 37339629 PMC10528006

[59] de Bruyn Kops A., and Knipe D.M. (1988). Formation of DNA replication structures in herpes virus-infected cells requires a viral DNA binding protein. Cell 55, 857–868. 10.1016/0092-8674(88)90141-9. 2847874

[60] Quinlan M.P., Chen L.B., and Knipe D.M. (1984). The intranuclear location of a herpes simplex virus DNA-binding protein is determined by the status of viral DNA replication. Cell 36, 857–868. 10.1016/0092-8674(84)90035-7. 6323024

[61] Seyffert M., Georgi F., Tobler K., Bourqui L., Anfossi M., Michaelsen K., Vogt B., Greber U.F., and Fraefel C. (2021). The HSV-1 transcription factor ICP4 confers liquid-like properties to viral replication compartments. Int. J. Mol. Sci. 22, 4447. 10.3390/ijms22094447. 33923223 PMC8123221

[62] Taylor T.J., McNamee E.E., Day C., and Knipe D.M. (2003). Herpes simplex virus replication compartments can form by coalescence of smaller compartments. Virology 309, 232–247. 10.1016/s0042-6822(03)00107-7. 12758171

[63] Shu T., Mitra G., Alberts J., Viana M.P., Levy E.D., Hocky G.M., and Holt L.J. (2024). Mesoscale molecular assembly is favored by the active, crowded cytoplasm. PRX Life 2, 033001. 10.1103/PRXLife.2.033001. 40162127 PMC11952695

[64] Banerjee D.S., Chigumira T., Lackner R.M., Kratz J.C., Chenoweth D.M., Banerjee S., and Zhang H. (2024). Interplay of condensate material properties and chromatin heterogeneity governs nuclear condensate ripening. eLife 13. 10.7554/eLife.101777.1.

[65] Keizer V.I.P., Grosse-Holz S., Woringer M., Zambon L., Aizel K., Bongaerts M., Delille F., Kolar-Znika L., Scolari V.F., Hoffmann S., (2022). Live-cell micromanipulation of a genomic locus reveals interphase chromatin mechanics. Science 377, 489–495. 10.1126/science.abi9810. 35901134

[66] Stancil I.T., Michalski J.E., Hennessy C.E., Hatakka K.L., Yang I.V., Kurche J.S., Rincon M., and Schwartz D.A. (2022). Interleukin-6-dependent epithelial fluidization initiates fibrotic lung remodeling. Sci. Transl. Med. 14, eabo5254. 10.1126/scitranslmed.abo5254. 35857823 PMC9981332

[67] Byfield F.J., Eftekhari B., Kaymak-Loveless K., Mandal K., Li D., Wells R.G., Chen W., Brujic J., Bergamaschi G., Wuite G.J.L., (2025). Metabolically intact nuclei are fluidized by the activity of the chromatin remodeling motor BRG1. Biophys. J. 124, 494–507. 10.1016/j.bpj.2024.11.3322. 39616442 PMC11866952

[68] Basu S., Shukron O., Hall D., Parutto P., Ponjavic A., Shah D., Boucher W., Lando D., Zhang W., Reynolds N., (2023). Live-cell three-dimensional single-molecule tracking reveals modulation of enhancer dynamics by NuRD. Nat. Struct. Mol. Biol. 30, 1628–1639. 10.1038/s41594-023-01095-4. 37770717 PMC10643137

[69] Dembowski J.A., and DeLuca N.A. (2015). Selective Recruitment of Nuclear Factors to Productively Replicating Herpes Simplex Virus Genomes. PLoS Pathog. 11, e1004939. 10.1371/journal.ppat.1004939. 26018390 PMC4446364

[70] Tsuchida N., Ryder T., and Ohtsubo E. (1982). Nucleotide sequence of the oncogene encoding the p21 transforming protein of Kirsten murine sarcoma virus. Science 217, 937–939. 10.1126/science.6287573. 6287573

[71] Tsuchida N., and Uesugi S. (1981). Structure and functions of the Kirsten murine sarcoma virus genome: molecular cloning of biologically active Kirsten murine sarcoma virus DNA. J. Virol. 38, 720–727. 10.1128/JVI.38.2.720-727.1981. 6264139 PMC171202

[72] Stehelin D., Varmus H.E., Bishop J.M., and Vogt P.K. (1976). DNA related to the transforming gene(s) of avian sarcoma viruses is present in normal avian DNA. Nature 260, 170–173. 10.1038/260170a0. 176594

[73] Hunter T., and Sefton B.M. (1980). Transforming gene product of Rous sarcoma virus phosphorylates tyrosine. Proc. Natl. Acad. Sci. U. S. A. 77, 1311–1315. 10.1073/pnas.77.3.1311. 6246487 PMC348484

[74] Brugge J.S., and Erikson R.L. (1977). Identification of a transformation-specific antigen induced by an avian sarcoma virus. Nature 269, 346–348. 10.1038/269346a0. 198667

[75] Matlashewski G., Lamb P., Pim D., Peacock J., Crawford L., and Benchimol S. (1984). Isolation and characterization of a human p53 cDNA clone: expression of the human p53 gene. EMBO J. 3, 3257–3262. 10.1002/j.1460-2075.1984.tb02287.x. 6396087 PMC557846

[76] Finlay C.A., Hinds P.W., and Levine A.J. (1989). The p53 proto-oncogene can act as a suppressor of transformation. Cell 57, 1083–1093. 10.1016/0092-8674(89)90045-7. 2525423

[77] Linzer D.I., Maltzman W., and Levine A.J. (1979). The SV40 A gene product is required for the production of a 54,000 MW cellular tumor antigen. Virology 98, 308–318. 10.1016/0042-6822(79)90554-3. 228475

[78] Vogt P.K. (2012). Retroviral oncogenes: a historical primer. Nat. Rev. Cancer 12, 639–648. 10.1038/nrc3320. 22898541 PMC3428493

[79] DeLuca N.A., and Schaffer P.A. (1988). Physical and functional domains of the herpes simplex virus transcriptional regulatory protein ICP4. J. Virol. 62, 732–743. 10.1128/jvi.62.3.732-743.1988. 2828668 PMC253626

[80] Shepard A.A., Imbalzano A.N., and DeLuca N.A. (1989). Separation of primary structural components conferring autoregulation, transactivation, and DNA-binding properties to the herpes simplex virus transcriptional regulatory protein ICP4. J. Virol. 63, 3714–3728. 10.1128/jvi.63.9.3714-3728.1989. 2760981 PMC250963

[81] Zhang H., Aonbangkhen C., Tarasovetc E.V., Ballister E.R., Chenoweth D.M., and Lampson M.A. (2017). Optogenetic control of kinetochore function. Nat. Chem. Biol. 13, 1096–1101. 10.1038/nchembio.2456. 28805800 PMC5605432

[82] Tokunaga M., Imamoto N., and Sakata-Sogawa K. (2008). Highly inclined thin illumination enables clear single-molecule imaging in cells. Nat. Methods 5, 159–161. 10.1038/nmeth1171. 18176568

[83] Keegan S., Fenyö D., and Holt L.J. (2023). GEMspa: a Napari plugin for analysis of single particle tracking data. bioRxiv. 10.1101/2023.06.26.546612.

[84] Stringer C., Wang T., Michaelos M., and Pachitariu M. (2021). Cellpose: a generalist algorithm for cellular segmentation. Nat Methods 18, 100–106. 10.1038/s41592-020-01018-x. 33318659

[85] Tinevez J.-Y., Perry N., Schindelin J., Hoopes G.M., Reynolds G.D., Laplantine E., Bednarek S.Y., Shorte S.L., and Eliceiri K.W. (2017). TrackMate: An open and extensible platform for single-particle tracking. Methods 115, 80–90. 10.1016/j.ymeth.2016.09.016. 27713081

[86] Shivanandan A., Radenovic A., and Sbalzarini I.F. (2013). MosaicIA: an ImageJ/Fiji plugin for spatial pattern and interaction analysis. BMC Bioinformatics 14, 349. 10.1186/1471-2105-14-349. 24299066 PMC4219334

[87] Schindelin J., Arganda-Carreras I., Frise E., Kaynig V., Longair M., Pietzsch T., Preibisch S., Rueden C., Saalfeld S., Schmid B., (2012). Fiji: an open-source platform for biological-image analysis. Nat Methods 9, 676–682. 10.1038/nmeth.2019. 22743772 PMC3855844

