# Supplementary material for: Herpes simplex virus-1 fluidizes the nucleus enabling condensate formation
Source: bioRxiv. 2025 Jun 21:2025.06.20.660750. Preprint. [Version 1] doi: 10.1101/2025.06.20.660750 (PMC12262588; doi:10.1101/2025.06.20.660750)
Supplement: Supplement 1 [file media-1.pdf]

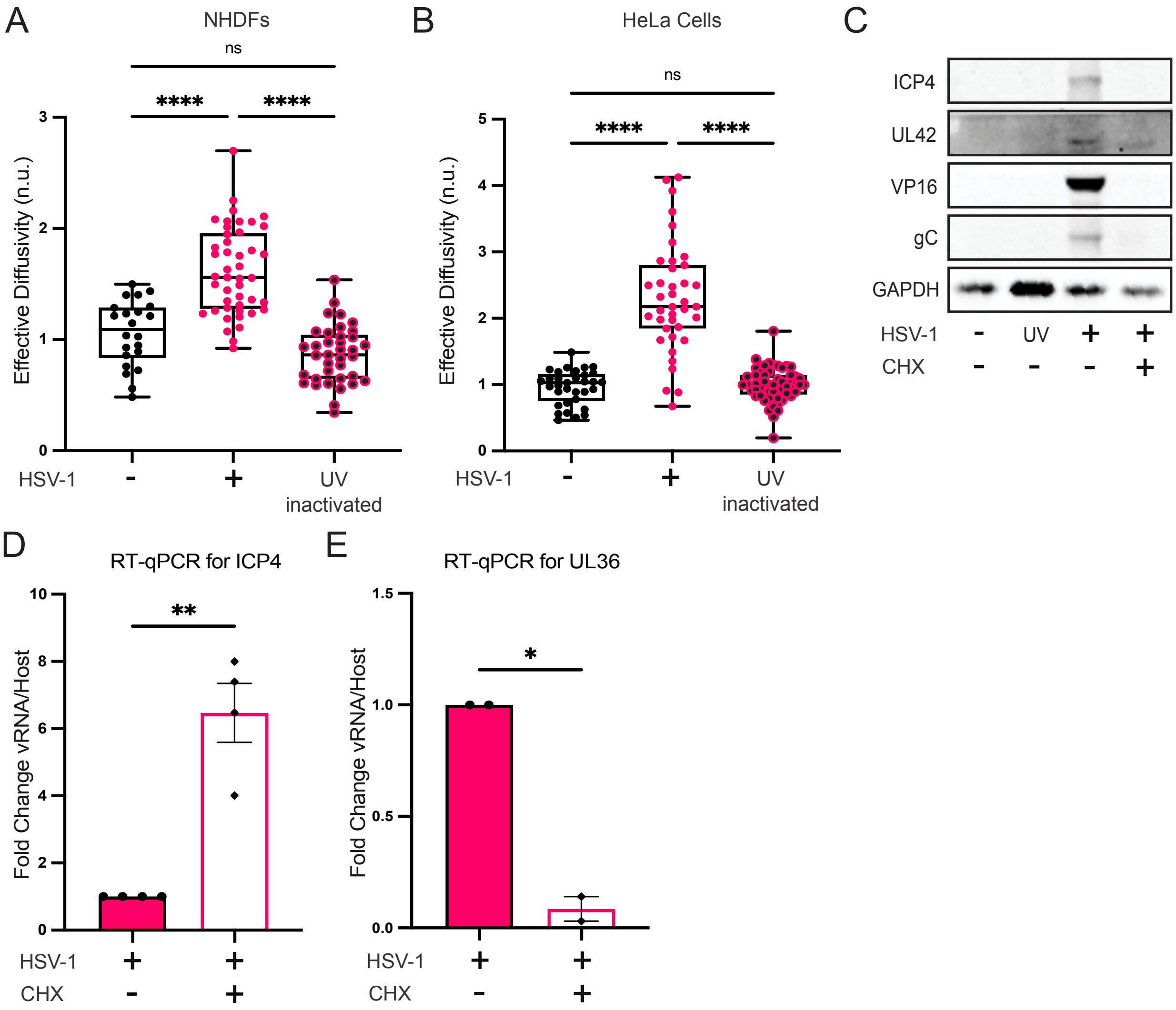

A
NHDFs
ns
****
****
Effective Diffusivity (n.u.)
0
1
2
3
HSV-1
-
+
UV
inactivated
B
HeLa Cells
ns
****
****
Effective Diffusivity (n.u.)
0
1
2
3
4
5
HSV-1
-
+
UV
inactivated
C
ICP4
UL42
VP16
gC
GAPDH
HSV-1
-
UV
+
+
CHX
-
-
-
+
D
RT-qPCR for ICP4
**
Fold Change vRNA/Host
0
2
4
6
8
10
HSV-1
+
+
CHX
-
+
E
RT-qPCR for UL36
*
Fold Change vRNA/Host
0.0
0.5
1.0
1.5
HSV-1
+
+
CHX
-
+

Supplemental Figure 1 (Supplement to Figure 1): Nuclear diffusivity increases during HSV-1 infection, dependent on new protein synthesis. (A) NHDFs transduced with nucGEMs were infected with HSV-1 KOS or UV-inactivated HSV-1 KOS at MOI =5. At 9 hpi, cells were imaged at 100 Hz and nucGEMs were tracked. Each point on the graph represents the median effective diffusion for all tracks in one cell. $n>40$; $N\geq3$ biological replicates. Statistical comparisons were performed using a Kruskal-Wallis test. (B) HeLa cells transduced with nucGEMs were infected, imaged, and analyzed as in panel A. $n>64$; $N\geq3$ biological replicates. (C) Immunoblot against immediate early, early, and late viral proteins in NHDFs 9 hpi with WT HSV-1, UV-inactivated HSV-1 (UV), or HSV-1 with cycloheximide (CHX). For CHX data, cells were pretreated for 30 minutes with cycloheximide (CHX) at 15 μg/mL then infected with HSV-1 KOS at an MOI of 5. Immunoblot is representative of N=3 biological replicates. (D) Total RNA was isolated from NHDFs at 9 hpi with HSV-1 KOS either treated with CHX as in panel C or left untreated. RT-qPCR analysis was performed for HSV-1 immediate early gene ICP4. $n>6$; N=3. Statistical comparisons were performed using a Kruskal-Wallis test. (E) Total RNA was isolated as in panel D. RT-qPCR analysis was performed for HSV-1 late gene UL36, $n>4$; N=2. Statistical comparisons were performed using a Kruskal-Wallis test. * signifies $p<0.05$, ** signifies $p<0.01$, **** signifies $p<0.0001$.

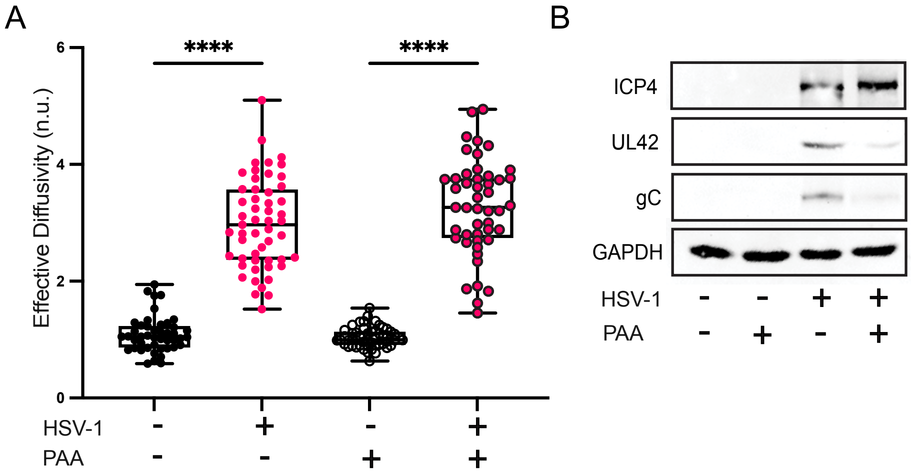
A
B
6
4
2
0
Effective Diffusivity (n.u.)
****
****
HSV-1
PAA
ICP4
UL42
gC
GAPDH
HSV-1
PAA

Supplemental Figure 2 (Supplement to Figure 2): HSV-1 infection fluidizes the nucleus independent of viral DNA synthesis, chromatin margination, and nuclear volume changes. (A) HeLa cells expressing nucGEMs were treated with 300 μg/mL phosphonoacetic acid (PAA) and were either left uninfected or infected with WT KOS HSV-1 at an MOI of 5. At 9 hpi, GEM movies and nuclei images were collected and analyzed. Statistical comparisons were performed using a two-way ANOVA. $n>91$; $N \geq 3$ biological replicates. (B) Immunoblot analysis to detect ICP4 (immediate-early), UL42 (early), and gC (late) viral protein expression in NHDFs infected for 9 h with WT HSV-1 treated with or without 300 μg/mL PAA. Immunoblot data is representative of N=3 biological replicates. **** signifies $p<0.0001$.

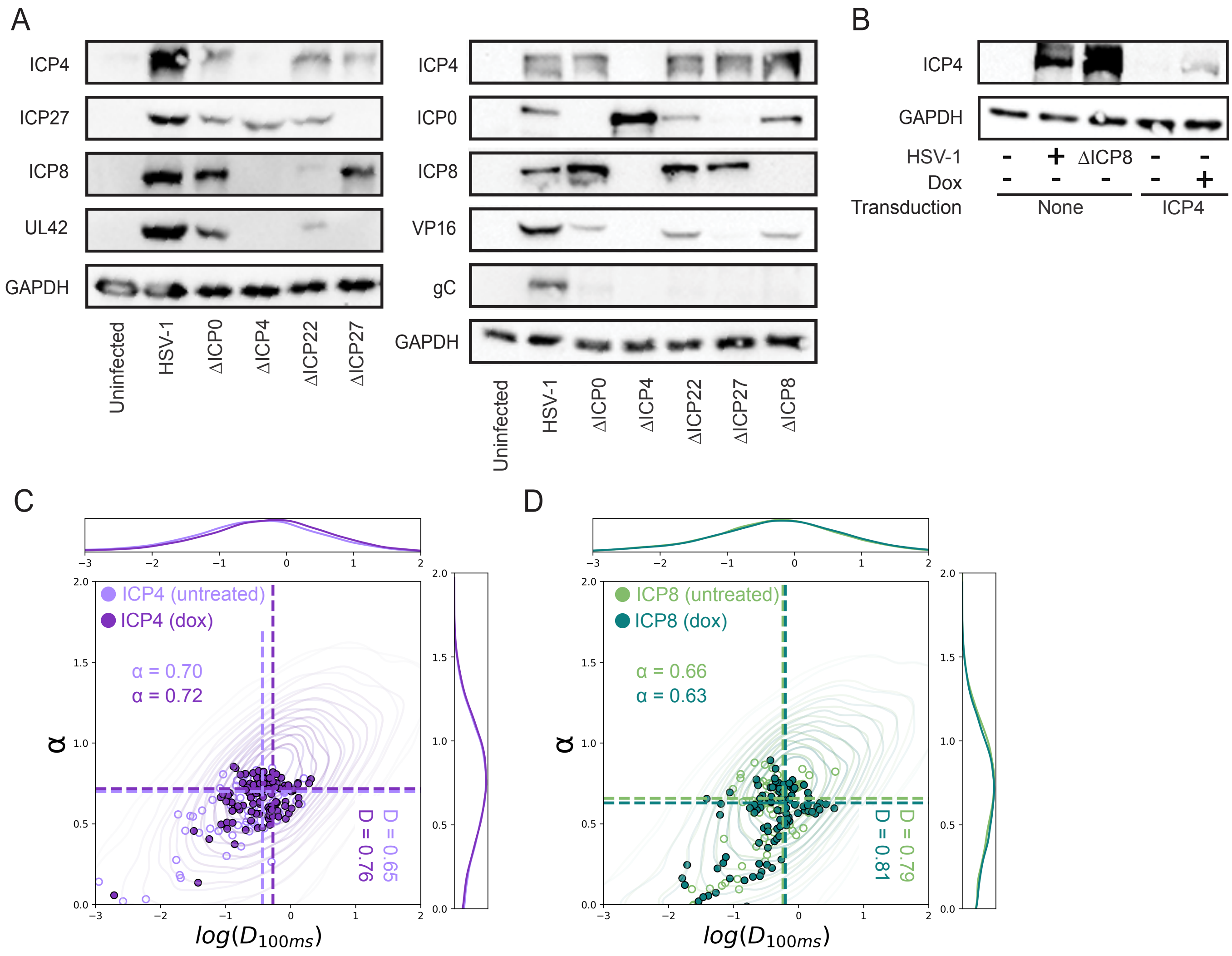
A
ICP4
ICP27
ICP8
UL42
GAPDH
Uninfected
HSV-1
ΔICP0
ΔICP4
ΔICP22
ΔICP27
ICP4
ICP0
ICP8
VP16
gC
GAPDH
Uninfected
HSV-1
ΔICP0
ΔICP4
ΔICP22
ΔICP27
ΔICP8
B
ICP4
GAPDH
HSV-1
Dox
Transduction
ΔICP8
None
ICP4
C
ICP4 (untreated)
ICP4 (dox)
α = 0.70
α = 0.72
D = 0.65
D = 0.76
α
log(D100ms)
D
ICP8 (untreated)
ICP8 (dox)
α = 0.66
α = 0.63
D = 0.79
D = 0.81
α
log(D100ms)

Supplemental Figure 3 (Supplement to Figure 3): HSV-1 immediate early protein ICP4 is necessary to fluidize the infected cell nucleus and sufficient to fluidize the nucleus of uninfected cells. (A) NHDFs were infected with WT KOS HSV-1, ∆ICP4 HSV-1, ∆ICP22 HSV-1, ∆ICP27 HSV-1, or ∆ICP8 HSV-1 at an MOI of 5, or with ∆ICP0 HSV-1 at an MOI of 10. Total protein was collected at 9 hpi, fractionated by SDS-PAGE, and analyzed by immunoblotting against the antibodies shown. GAPDH served as the loading control. Data is representative of N=3 biological replicates. (B) NHDFs were transduced as described (Methods) with a lentivirus expressing codon-optimized ICP4 under a tet-promoter or left untransduced. Untransduced cells were then infected with WT HSV-1 KOS or ∆ICP8 HSV-1 at an MOI of 5 for 9 h; in transduced cells ICP4 protein expression was induced for 9 h with doxycycline. At 9 hpt total protein was collected and processed as in panel A. Data is representative of N=3 biological replicates. (C) Graph of anomalous exponent (alpha) compared to the log effective diffusion of tracks in the presence (purple) and absence (grey) of ICP4 induction in NHDFs. Data is pooled from N=4 biological replicates. Frequency maps along each axis represent the number of cells with a given value. (D) Graph of anomalous exponent (alpha) compared to the log effective diffusion of tracks in the presence (green) and absence (grey) of ICP8 induction in NHDFs. Data is pooled from N=4 biological replicates. Frequency maps along each axis represent the number of cells with a given value. Statistical analysis (C, D) available in supplemental table 4.

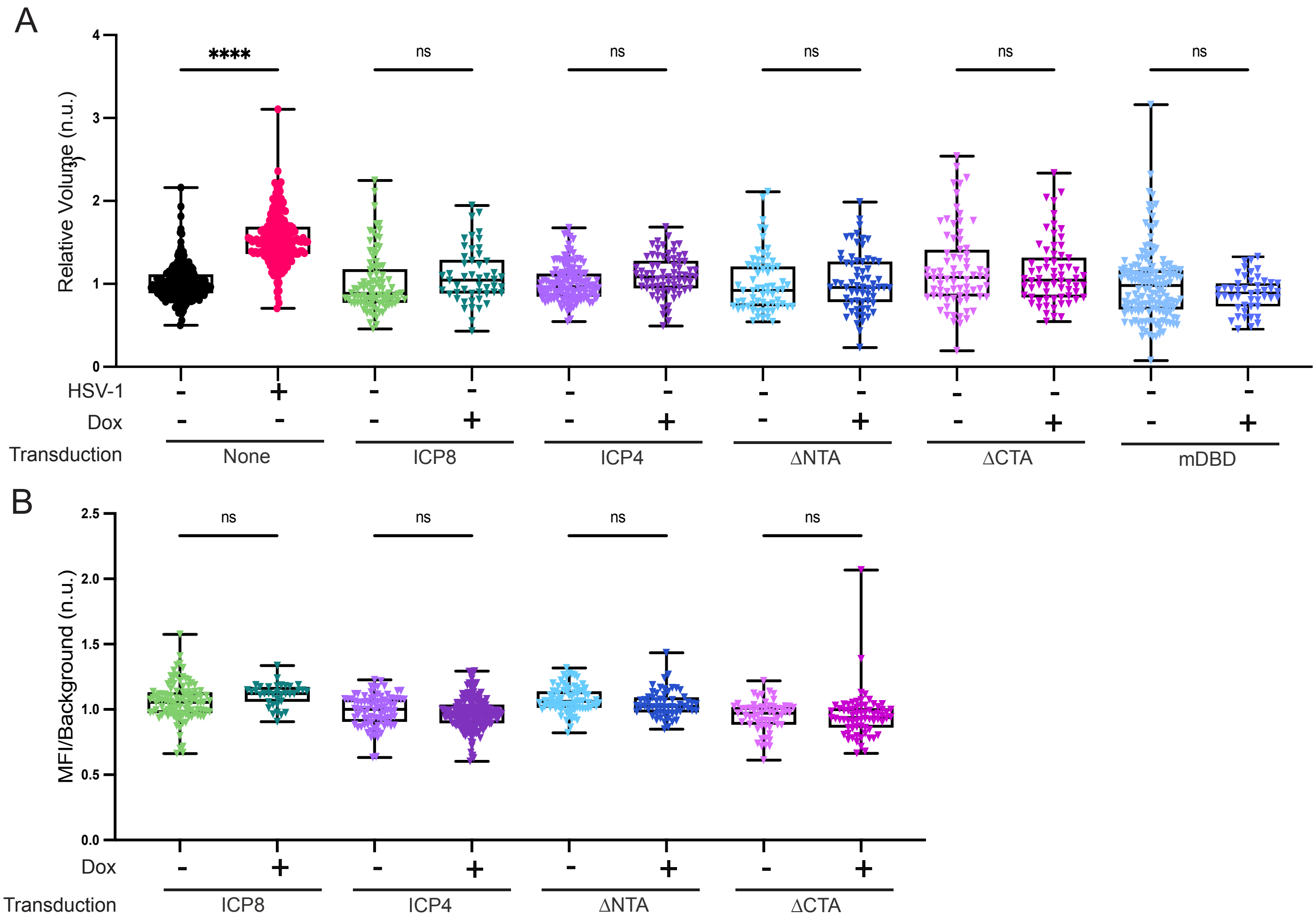
A
Relative Volume (n.u.)
0
1
2
3
4
****
ns
ns
ns
ns
ns
HSV-1
Dox
Transduction
None
ICP8
ICP4
ΔNTA
ΔCTA
mDBD
B
MFI/Background (n.u.)
0.0
0.5
1.0
1.5
2.0
2.5
ns
ns
ns
ns
Dox
Transduction
ICP8
ICP4
ΔNTA
ΔCTA

Supplemental Figure 4 (Supplement to Figure 4): Both N-terminal and C-terminal domains of ICP4 are required to increase nucGEM diffusivity in uninfected cells. (A) NHDFs expressing nucGEMs were transduced as described (Methods) with lentivirus expressing codon-optimized ICP4, ICP8, or ICP4 mutants described in (**Fig. 4A**) under a tet-promoter. Cells were either infected with WT HSV-1 KOS at an MOI of 5 or protein expression was induced for 9 h. At 9 hpt, cells were stained using vital stain SiR-DNA to visualize the host nucleus. Z-stacks were obtained, nuclear masks were created using cellpose, and relative nuclear volume of cells was calculated with Foci-Counting (Methods). $n>90$; $N\geq3$ biological replicates. (B) NHDFs were transduced as in panel A. Protein expression was induced for 7 h, then cells were incubated for 2 h with the nucleoside analog 5-ethynyl uridine (5EU). At 9 hpt cells were fixed using PFA and click chemistry was performed. Cells were additionally probed for ICP4 or ICP8 via IF. Cells were verified for ICP4 or ICP8 expression and mean fluorescence intensity (MFI) was calculated using Foci-Counting, then normalized to background (Methods). $n>99$; $N\geq3$ biological replicates. All statistical comparisons were performed using a Kruskal-Wallis test. **** signifies $p<0.0001$.

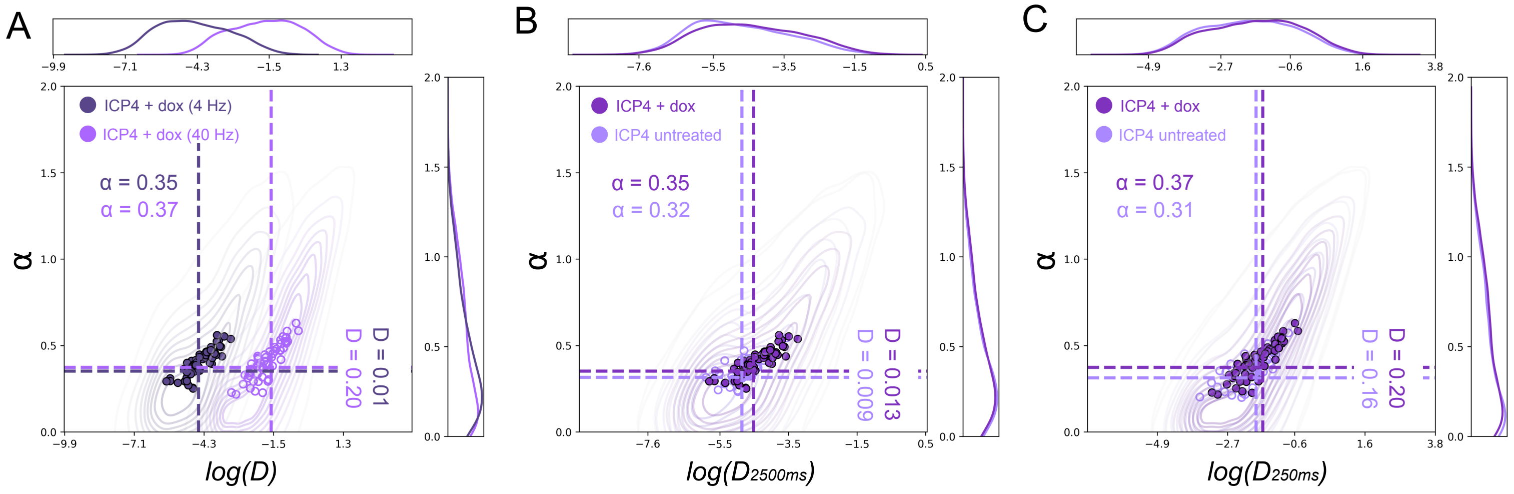
A
B
C
ICP4 + dox (4 Hz)
ICP4 + dox (40 Hz)
α = 0.35
α = 0.37
D = 0.01
D = 0.20
log(D)
α
ICP4 + dox
ICP4 untreated
α = 0.35
α = 0.32
D = 0.013
D = 0.009
log(D2500ms)
α = 0.37
α = 0.31
D = 0.20
D = 0.16
log(D250ms)

Supplemental Figure 5 (Supplement to Figure 5): Movement of bound histones increases during ICP4 induction and during HSV-1 infection dependent on the presence of ICP4. (A) Graph of anomalous exponent (alpha) compared to the log effective diffusion of Halo-H2A tracks at 4 Hz (purple) and 40 Hz (grey) of ICP4 induction in NHDFs. (B) Graph of anomalous exponent (alpha) compared to the log effective diffusion of Halo-H2A tracks at 4 Hz in the presence (purple) and absence (grey) of ICP4 induction in NHDFs. (C) Graph of anomalous exponent (alpha) compared to the log effective diffusion of Halo-H2A tracks at 40 Hz in the presence (purple) and absence (grey) of ICP4 induction in NHDFs. Frequency maps along each axis represent the number of cells with a given value. All data is pooled from N=4 biological replicates. Statistical analysis available in supplemental table 5.

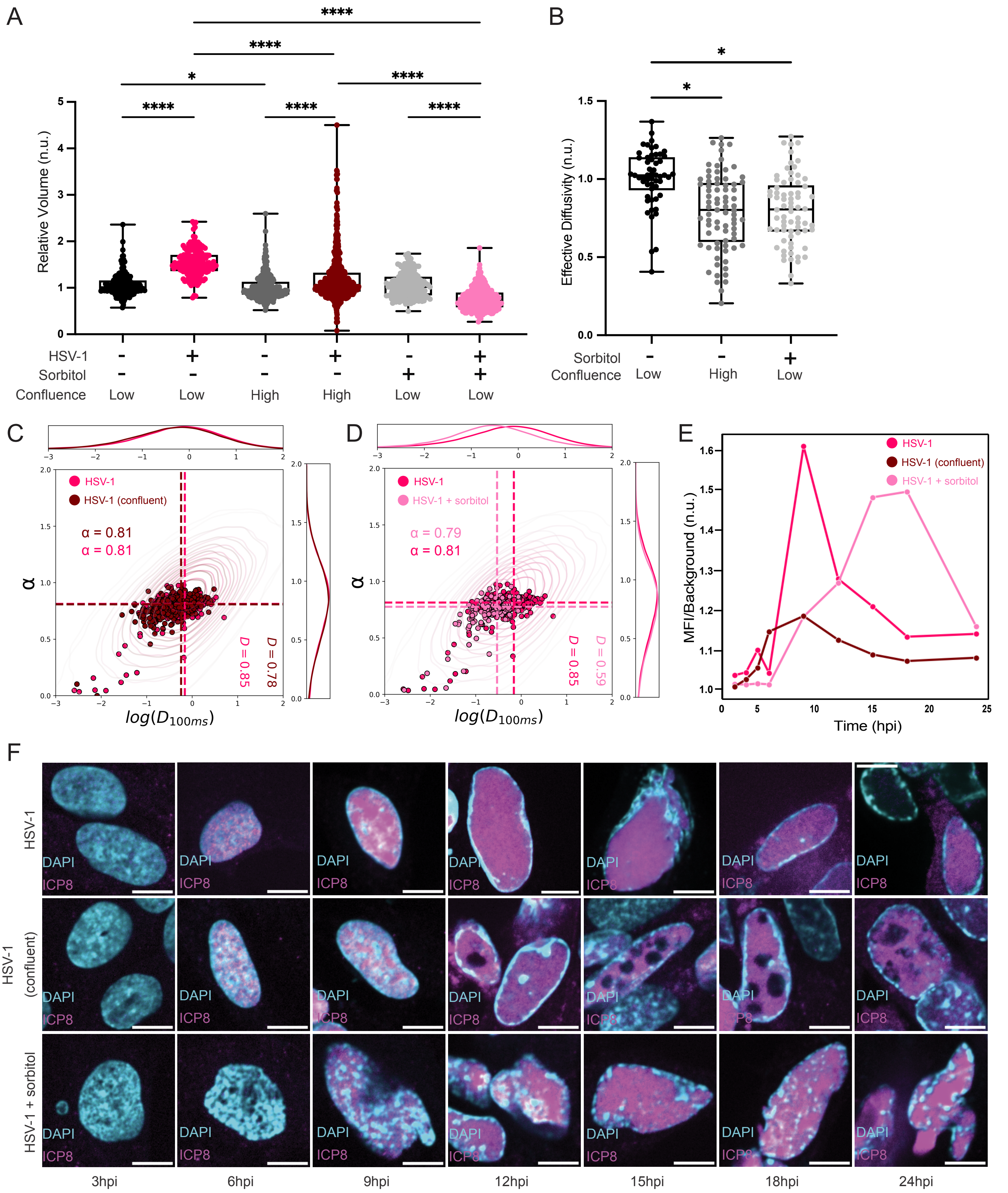

A
B
C
D
E
F
****
*
Relative Volume (n.u.)
HSV-1
Sorbitol
Confluence
Low
High
Effective Diffusivity (n.u.)
HSV-1
HSV-1 (confluent)
HSV-1 + sorbitol
α = 0.81
α = 0.79
D = 0.85
D = 0.78
D = 0.59
α
log(D100ms)
MFI/Background (n.u.)
Time (hpi)
DAPI
ICP8
3hpi
6hpi
9hpi
12hpi
15hpi
18hpi
24hpi

Supplemental Figure 6 (Supplement to Figure 6): Preventing nuclear fluidization disrupts viral replication compartment formation and decreases infectious virus production. (A) NHDFs transduced with nucGEMs were grown to a confluent monolayer (High), then infected with WT HSV-1 at MOI = 5. NHDFs transduced with nucGEMs were grown to 70% confluency, then infected with WT HSV-1 at MOI = 5 (Low). At 2.5 hpi, cells were left untreated or were treated with 150 mM sorbitol. At 9 hpi cells were stained using vital stain SiR-DNA to visualize the host nucleus. Z-stacks were obtained, nuclear masks were created using cellpose, and relative nuclear volume of cells was calculated with Foci-Counting (Methods). Statistical comparisons were performed using a two-way ANOVA with Bonferroni's correction. n>353; N≥3 biological replicates. (B) NHDFs transduced with nucGEMs were grown to a confluent monolayer (High) or were grown to 70% confluency (Low) and either left untreated or were treated with 150 mM sorbitol. nucGEM diffusivity was measured at 6.5 h as previously described (Methods). Statistical comparisons were performed using a Kruskal-Wallis test. n>136; N≥3 biological replicates. (C) Graph of anomalous exponent (alpha) compared to the log effective diffusion of tracks during WT HSV-1 infection (HSV-1) vs. WT HSV-1 infection in confluent monolayers (HSV-1 (confluent)). Data is pooled from N=4 biological replicates. Frequency maps along each axis represent the number of cells with a given value. (D) Graph of anomalous exponent (alpha) compared to the log effective diffusion of tracks during WT HSV-1 infection (HSV-1) vs. WT HSV-1 infection with sorbitol treatment as described in (B) (HSV-1 + sorbitol). Data is pooled from N=4 biological replicates. Frequency maps along each axis represent the number of cells with a given value. (E) Stacked line graph of data in (**Fig. 6G**) for parallel visualization of all vRC kinetics under each condition. Median MFI across all replicates was plotted. (F) Representative immunofluorescence from cells in panel E. Statistical analysis (C, D) available in supplemental table 6. All experiments are at least N=3 biological replicates. IF images are representative of N=3 biological replicates. * signifies $p<0.05$, **** signifies $p<0.0001$.

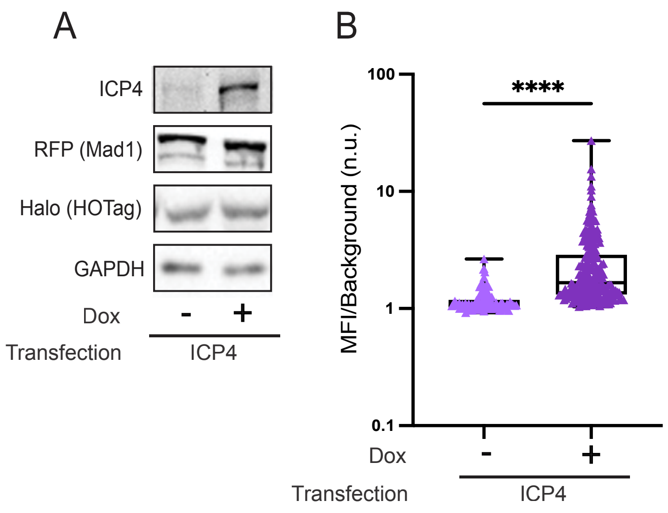
A
ICP4
RFP (Mad1)
Halo (HOTag)
GAPDH
Dox
-
+
Transfection
ICP4
B
100
10
1
0.1
MFI/Background (n.u.)
****
Dox
-
+
Transfection
ICP4

Supplemental Figure 7 (Supplement to Figure 7): Fluidization of the nucleus by ICP4 facilitates growth of artificial condensates. (A) Immunoblot against ICP4 and the components of the artificial condensate system as described (**Fig. 7A**, Methods). HeLa cells were transfected with the plasmid containing inducible codon-optimized ICP4 as well as the two plasmids forming the artificial condensate system as described (Methods). Cells were treated with 3 µg/mL doxycycline for 8 h or left untreated. At 8 hpt, all conditions were treated with 50 nM chemical dimerizer Trimethoprim-Fluorobenzamide-Halo ligand (TFH) for 1 h. After 1 h of TFH treatment, total protein was collected, fractionated by SDS-PAGE, and analyzed by immunoblotting using the antibodies shown. GAPDH was used as a loading control. Data is representative of N=3 biological replicates. (B) Cells were prepared and treated as in panel A. Following TFH treatment, cells were fixed for IF using PFA, then probed for ICP4. Cells were verified for ICP4 expression, nuclear masks were created using cellpose, and mean fluorescence intensity (MFI) of RFP per cell was calculated using Foci-Counting, then normalized to background (Methods). n>224; N=4 biological replicates. IF images are representative of N=3 biological replicates. Statistical comparisons were performed using a Mann-Whitney test. **** signifies $p<0.0001$.

Supplemental Table 1: Statistical analysis for Fig. 1E

Welch's t-test of Log(D_{100ms}) and α (alpha)							
**variable**	**group1**	**group2**	**mean_group1**	**mean_group2**	**t_statistic**	**p_value**	**significant**
**log_D**	Uninfected	HSV-1	-0.818024253	-0.129086091	-141.1539303	p<0.0001	TRUE
**alpha**	Uninfected	HSV-1	0.692879712	0.796449879	-54.85629231	p<0.0001	TRUE

Supplemental Table 2: Statistical analysis for Fig. 6G

Two-way ANOVA of vRC MFI (n.u.)

ANOVA table	SS (Type III)	DF	MS	F (DFn, DFd)	P value
Row Factor	27.45	591	0.04645	F (591, 16038) = 0.8521	0.9956
Column Factor	293	26	11.27	F (26, 16038) = 206.7	<0.0001
Residual	874.4	16038	0.05452		

Multiple Comparison of Mean vRC MFI (n.u.) — Tukey HSD, FWER=0.05

Group1	Group2	MeanDiff	p-adj	lower	upper	reject
HSV-1 3hpi	HSV-1 4hpi	-0.02911	0.9968	-0.09324	0.03502	FALSE
HSV-1 3hpi	HSV-1 5hpi	-0.07935	0.0007	-0.1413	-0.01744	TRUE
HSV-1 3hpi	HSV-1 6hpi	-0.2111	<0.0001	-0.2672	-0.1549	TRUE
HSV-1 3hpi	HSV-1 9hpi	-0.519	<0.0001	-0.5744	-0.4635	TRUE
HSV-1 3hpi	HSV-1 12hpi	-0.1935	<0.0001	-0.2559	-0.1312	TRUE
HSV-1 3hpi	HSV-1 15hpi	-0.121	<0.0001	-0.1801	-0.06184	TRUE
HSV-1 3hpi	HSV-1 18hpi	-0.08148	0.0011	-0.1464	-0.01658	TRUE
HSV-1 3hpi	HSV-1 24hpi	-0.1644	<0.0001	-0.2273	-0.1015	TRUE
HSV-1 + sorbitol 3hpi	HSV-1 + sorbitol 4hpi	-0.00243	>0.9999	-0.06788	0.06301	FALSE
HSV-1 + sorbitol 3hpi	HSV-1 + sorbitol 5hpi	-0.00205	>0.9999	-0.06718	0.06308	FALSE
HSV-1 + sorbitol 3hpi	HSV-1 + sorbitol 6hpi	-0.08551	<0.0001	-0.1426	-0.02838	TRUE
HSV-1 + sorbitol 3hpi	HSV-1 + sorbitol 9hpi	-0.2687	<0.0001	-0.3242	-0.2133	TRUE
HSV-1 + sorbitol 3hpi	HSV-1 + sorbitol 12hpi	-0.237	<0.0001	-0.3006	-0.1735	TRUE
HSV-1 + sorbitol 3hpi	HSV-1 + sorbitol 15hpi	-0.2861	<0.0001	-0.348	-0.2242	TRUE
HSV-1 + sorbitol 3hpi	HSV-1 + sorbitol 18hpi	-0.3592	<0.0001	-0.4243	-0.2942	TRUE
HSV-1 + sorbitol 3hpi	HSV-1 + sorbitol 24hpi	-0.5721	<0.0001	-0.6409	-0.5032	TRUE
HSV-1 (confluent) 3hpi	HSV-1 (confluent) 4hpi	-0.02045	>0.9999	-0.07796	0.03705	FALSE
HSV-1 (confluent) 3hpi	HSV-1 (confluent) 5hpi	-0.04067	0.6386	-0.09801	0.01667	FALSE
HSV-1 (confluent) 3hpi	HSV-1 (confluent) 6hpi	-0.2048	<0.0001	-0.256	-0.1536	TRUE
HSV-1 (confluent) 3hpi	HSV-1 (confluent) 9hpi	-0.2425	<0.0001	-0.291	-0.1939	TRUE
HSV-1 (confluent) 3hpi	HSV-1 (confluent) 12hpi	-0.1546	<0.0001	-0.2064	-0.1028	TRUE
HSV-1 (confluent) 3hpi	HSV-1 (confluent) 15hpi	-0.119	<0.0001	-0.1713	-0.06663	TRUE
HSV-1 (confluent) 3hpi	HSV-1 (confluent) 18hpi	-0.1107	<0.0001	-0.1634	-0.05804	TRUE
HSV-1 (confluent) 3hpi	HSV-1 (confluent) 24hpi	-0.1056	<0.0001	-0.1577	-0.05343	TRUE
HSV-1 3hpi	HSV-1 + sorbitol 3hpi	0.03178	0.9927	-0.03433	0.09789	FALSE
HSV-1 3hpi	HSV-1 (confluent) 3hpi	0.0254	0.9995	-0.03704	0.08785	FALSE
HSV-1 4hpi	HSV-1 + sorbitol 4hpi	0.05845	0.123	-0.004938	0.1218	FALSE
HSV-1 4hpi	HSV-1 (confluent) 4hpi	0.03406	0.9393	-0.02563	0.09375	FALSE
HSV-1 5hpi	HSV-1 + sorbitol 5hpi	0.1091	<0.0001	0.04821	0.1699	TRUE
HSV-1 5hpi	HSV-1 (confluent) 5hpi	0.06408	0.009	0.007098	0.1211	TRUE
HSV-1 6hpi	HSV-1 + sorbitol 6hpi	0.1573	<0.0001	0.1126	0.202	TRUE
HSV-1 6hpi	HSV-1 (confluent) 6hpi	0.03165	0.5697	-0.01158	0.07487	FALSE
HSV-1 9hpi	HSV-1 + sorbitol 9hpi	0.282	<0.0001	0.2404	0.3236	TRUE
HSV-1 9hpi	HSV-1 (confluent) 9hpi	0.3019	<0.0001	0.2629	0.3408	TRUE
HSV-1 12hpi	HSV-1 + sorbitol 12hpi	-0.01173	>0.9999	-0.07102	0.04756	FALSE
HSV-1 12hpi	HSV-1 (confluent) 12hpi	0.06438	0.0013	0.01269	0.1161	TRUE
HSV-1 15hpi	HSV-1 + sorbitol 15hpi	-0.1334	<0.0001	-0.187	-0.07974	TRUE
HSV-1 15hpi	HSV-1 (confluent) 15hpi	0.02737	0.9358	-0.02034	0.07508	FALSE
HSV-1 18hpi	HSV-1 + sorbitol 18hpi	-0.246	<0.0001	-0.3096	-0.1823	TRUE
HSV-1 18hpi	HSV-1 (confluent) 18hpi	-0.00383	>0.9999	-0.05954	0.05188	FALSE
HSV-1 24hpi	HSV-1 + sorbitol 24hpi	-0.3759	<0.0001	-0.4417	-0.3101	TRUE
HSV-1 24hpi	HSV-1 (confluent) 24hpi	0.0842	<0.0001	0.03187	0.1365	TRUE

Supplemental Table 3: Statistical analysis of Fig. 7C, Fig. 7D, Fig. 7E

Independent t-test of condensate count per cell							
**Group 1**	**Group 2**	**t/t0**	**Group 1 Mean**	**Group 2 Mean**	**t-statistic**	**p-value**	**Reject**
ICP4 + Dox	ICP4 Untreated	0	2.352941176	1.88	1.168882456	0.247796811	FALSE
ICP4 + Dox	ICP4 Untreated	1	3.189189189	2.62962963	0.992582707	0.325111173	FALSE
ICP4 + Dox	ICP4 Untreated	2	4.4	4.419354839	-0.022541243	0.982090981	FALSE
ICP4 + Dox	ICP4 Untreated	3	4.960784314	6.578947368	-1.553284158	0.125219085	FALSE
ICP4 + Dox	ICP4 Untreated	4	5.866666667	7.234042553	-1.38395612	0.169720645	FALSE
ICP4 + Dox	ICP4 Untreated	5	7.030769231	8.019230769	-1.021024169	0.309651037	FALSE
ICP4 + Dox	ICP4 Untreated	6	8.060606061	8.298245614	-0.246283946	0.805904149	FALSE
ICP4 + Dox	ICP4 Untreated	7	8.909090909	8.694915254	0.220407351	0.825934144	FALSE
ICP4 + Dox	ICP4 Untreated	8	8.956521739	8.868852459	0.090373431	0.928136641	FALSE
ICP4 + Dox	ICP4 Untreated	9	9.242857143	9.161290323	0.084227291	0.933009573	FALSE
ICP4 + Dox	ICP4 Untreated	10	9.464788732	9.285714286	0.182816459	0.855230248	FALSE
ICP4 + Dox	ICP4 Untreated	11	9.662162162	9.555555556	0.107943636	0.914206165	FALSE
ICP4 + Dox	ICP4 Untreated	12	9.723684211	9.857142857	-0.135674962	0.892285912	FALSE
ICP4 + Dox	ICP4 Untreated	13	9.658227848	10.23809524	-0.595260444	0.552676108	FALSE
ICP4 + Dox	ICP4 Untreated	14	9.962025316	10.47619048	-0.514700004	0.60760846	FALSE
ICP4 + Dox	ICP4 Untreated	15	10.16049383	10.71428571	-0.548998339	0.58391125	FALSE
ICP4 + Dox	ICP4 Untreated	16	10.37037037	10.67692308	-0.295592386	0.767989725	FALSE
ICP4 + Dox	ICP4 Untreated	17	10.63855422	10.90769231	-0.257107702	0.797481831	FALSE
ICP4 + Dox	ICP4 Untreated	18	11	11.12121212	-0.111104884	0.911694745	FALSE
ICP4 + Dox	ICP4 Untreated	19	11.32142857	11.43939394	-0.105874899	0.915834159	FALSE
ICP4 + Dox	ICP4 Untreated	20	11.6	11.75757576	-0.141410222	0.887747277	FALSE
ICP4 + Dox	ICP4 Untreated	21	11.98823529	11.93939394	0.042904221	0.96583827	FALSE
ICP4 + Dox	ICP4 Untreated	22	12.43529412	12.24242424	0.163981623	0.869973873	FALSE
ICP4 + Dox	ICP4 Untreated	23	12.64705882	12.68181818	-0.029187114	0.976755656	FALSE
ICP4 + Dox	ICP4 Untreated	24	13.05882353	13.01515152	0.035728697	0.971548403	FALSE
ICP4 + Dox	ICP4 Untreated	25	13.50588235	13.34848485	0.12550164	0.900302986	FALSE
ICP4 + Dox	ICP4 Untreated	26	13.98823529	13.57575758	0.317165738	0.751580328	FALSE
ICP4 + Dox	ICP4 Untreated	27	14.28235294	13.84848485	0.32507335	0.745604611	FALSE
ICP4 + Dox	ICP4 Untreated	28	14.28235294	13.84848485	0.32507335	0.745604611	FALSE
ICP4 + Dox	ICP4 Untreated	29	14.28235294	13.84848485	0.32507335	0.745604611	FALSE
ICP4 + Dox	ICP4 Untreated	30	14.28235294	13.84848485	0.32507335	0.745604611	FALSE
**Independent t-test of R/R0**							
**Group 1**	**Group 2**	**t/t0**	**Group 1 Mean**	**Group 2 Mean**	**t-statistic**	**p-value**	**Reject**
ICP4 + Dox	ICP4 Untreated	0	1.174967089	0.778012096	5.198373393	5.24E-07	TRUE
ICP4 + Dox	ICP4 Untreated	1	1	1	-	-	-
ICP4 + Dox	ICP4 Untreated	2	1.537614063	1.863523798	-1.97756027	0.048969876	TRUE
ICP4 + Dox	ICP4 Untreated	3	2.230352467	2.16533689	0.359929475	0.719144548	FALSE
ICP4 + Dox	ICP4 Untreated	4	2.98419341	2.480592537	2.667519572	0.008042592	TRUE
ICP4 + Dox	ICP4 Untreated	5	3.31378591	2.650254102	3.274413685	0.001178208	TRUE
ICP4 + Dox	ICP4 Untreated	6	3.446338921	2.7887401	3.193641354	0.001552965	TRUE
ICP4 + Dox	ICP4 Untreated	7	3.662189308	2.767026316	4.348247686	1.87E-05	TRUE
ICP4 + Dox	ICP4 Untreated	8	3.740582452	2.78025408	4.584545131	6.67E-06	TRUE
ICP4 + Dox	ICP4 Untreated	9	3.806296834	2.789549812	5.000343226	9.68E-07	TRUE
ICP4 + Dox	ICP4 Untreated	10	3.789500958	2.832477584	4.566930928	7.23E-06	TRUE
ICP4 + Dox	ICP4 Untreated	11	3.74619641	2.822884107	4.448149286	1.23E-05	TRUE
ICP4 + Dox	ICP4 Untreated	12	3.789499984	2.841257303	4.561422549	7.40E-06	TRUE

ICP4 + Dox	ICP4 Untreated	13	3.927816501	2.866956381	4.843163236	2.05E-06	TRUE
ICP4 + Dox	ICP4 Untreated	14	3.912022442	2.856061222	4.790705411	2.62E-06	TRUE
ICP4 + Dox	ICP4 Untreated	15	3.920897003	2.83438289	5.066593989	7.07E-07	TRUE
ICP4 + Dox	ICP4 Untreated	16	3.932064087	2.840346632	5.082926439	6.55E-07	TRUE
ICP4 + Dox	ICP4 Untreated	17	3.836400105	2.860668019	4.503137618	9.58E-06	TRUE
ICP4 + Dox	ICP4 Untreated	18	3.778740712	2.858336512	4.322670721	2.10E-05	TRUE
ICP4 + Dox	ICP4 Untreated	19	3.75108435	2.870119005	4.077457992	5.88E-05	TRUE
ICP4 + Dox	ICP4 Untreated	20	3.75185213	2.82494463	4.371839811	1.70E-05	TRUE
ICP4 + Dox	ICP4 Untreated	21	3.767321397	2.776276119	4.656496913	4.82E-06	TRUE
ICP4 + Dox	ICP4 Untreated	22	3.808600949	2.820064372	4.679783807	4.35E-06	TRUE
ICP4 + Dox	ICP4 Untreated	23	3.77975886	2.808503929	4.612313841	5.91E-06	TRUE
ICP4 + Dox	ICP4 Untreated	24	3.785988717	2.850876804	4.357800157	1.82E-05	TRUE
ICP4 + Dox	ICP4 Untreated	25	3.76613203	2.844883132	4.29399812	2.38E-05	TRUE
ICP4 + Dox	ICP4 Untreated	26	3.779103641	2.873360563	4.146192261	4.43E-05	TRUE
ICP4 + Dox	ICP4 Untreated	27	3.686014666	2.778421836	4.37230407	1.71E-05	TRUE
ICP4 + Dox	ICP4 Untreated	28	3.63253516	2.786234598	3.909482604	0.000115181	TRUE
ICP4 + Dox	ICP4 Untreated	29	3.608946765	2.783536085	3.874770058	0.000132257	TRUE

Independent t-test of individual total condensate intensity

Group 1	Group 2	t/t0	Group 1 Mean	Group 2 Mean	t-statistic	p-value	Reject
ICP4 + Dox	ICP4 Untreated	0	1.819160393	0.753261776	3.163439386	0.001932449	TRUE
ICP4 + Dox	ICP4 Untreated	1	1	1	-	-	-
ICP4 + Dox	ICP4 Untreated	2	4.81805612	7.106141591	-1.347764996	0.179021326	FALSE
ICP4 + Dox	ICP4 Untreated	3	10.0273899	9.503824831	0.281310687	0.778671689	FALSE
ICP4 + Dox	ICP4 Untreated	4	16.61818744	12.52824829	1.93421598	0.053993953	FALSE
ICP4 + Dox	ICP4 Untreated	5	21.52416715	15.09167394	2.480297818	0.013659091	TRUE
ICP4 + Dox	ICP4 Untreated	6	23.18740802	17.59375055	1.968003429	0.049968654	TRUE
ICP4 + Dox	ICP4 Untreated	7	26.33164749	17.78268277	2.942847511	0.003497275	TRUE
ICP4 + Dox	ICP4 Untreated	8	27.83867205	18.88759448	2.852947832	0.004630879	TRUE
ICP4 + Dox	ICP4 Untreated	9	28.87550429	18.94321931	3.269722134	0.001198657	TRUE
ICP4 + Dox	ICP4 Untreated	10	29.31237069	19.93860843	2.846942948	0.004717979	TRUE
ICP4 + Dox	ICP4 Untreated	11	28.80452435	20.68667919	2.368968523	0.018507286	TRUE
ICP4 + Dox	ICP4 Untreated	12	30.07036799	20.7571277	2.774789248	0.005872284	TRUE
ICP4 + Dox	ICP4 Untreated	13	33.00525778	22.02540707	2.944086816	0.003495146	TRUE
ICP4 + Dox	ICP4 Untreated	14	33.40268396	22.07812092	2.978784627	0.003136381	TRUE
ICP4 + Dox	ICP4 Untreated	15	33.76764025	21.75813273	3.213572314	0.001453501	TRUE
ICP4 + Dox	ICP4 Untreated	16	33.71064882	22.06184513	3.114062853	0.002028252	TRUE
ICP4 + Dox	ICP4 Untreated	17	33.00281889	22.60488145	2.662138743	0.008188252	TRUE
ICP4 + Dox	ICP4 Untreated	18	32.06660885	22.55331309	2.447611675	0.014947546	TRUE
ICP4 + Dox	ICP4 Untreated	19	31.33033865	22.8993569	2.200201913	0.028606587	TRUE
ICP4 + Dox	ICP4 Untreated	20	31.64387065	21.98773332	2.563329469	0.01086356	TRUE
ICP4 + Dox	ICP4 Untreated	21	32.34620488	21.48450489	2.831936128	0.004935734	TRUE
ICP4 + Dox	ICP4 Untreated	22	32.73364426	21.88897094	2.901112782	0.003988091	TRUE
ICP4 + Dox	ICP4 Untreated	23	31.96076	22.3114988	2.609179767	0.009535553	TRUE
ICP4 + Dox	ICP4 Untreated	24	31.8754685	23.55497277	2.161495999	0.031504136	TRUE
ICP4 + Dox	ICP4 Untreated	25	31.63797667	23.19155166	2.211753104	0.027784535	TRUE
ICP4 + Dox	ICP4 Untreated	26	31.74983522	23.64361409	2.056689243	0.040645811	TRUE
ICP4 + Dox	ICP4 Untreated	27	29.98339235	22.37634964	2.042355947	0.04208154	TRUE
ICP4 + Dox	ICP4 Untreated	28	29.97309399	22.98469628	1.785149295	0.075361739	FALSE
ICP4 + Dox	ICP4 Untreated	29	29.54011714	22.83062444	1.748667137	0.081503225	FALSE

Supplemental Table 4: Statistical analysis for Supplemental Fig. 3C, Supplemental Fig. 3D

Welch's T-test of Log(D_{100ms}) and α (alpha)							
**variable**	**group1**	**group2**	**mean_group1**	**mean_group2**	**t_statistic**	**p_value**	**significant**
**log_D**	ICP4_none	ICP4_dox	-0.507744888	-0.345600664	-22.13672406	<0.0001	TRUE
**aexp**	ICP4_none	ICP4_dox	0.672656438	0.69236511	-7.197036553	<0.0001	TRUE
**log_D**	ICP8_none	ICP8_dox	-0.283165239	-0.244901402	-4.492237037	<0.0001	TRUE
**aexp**	ICP8_none	ICP8_dox	0.625674373	0.602967593	6.590069373	<0.0001	TRUE

Supplemental Table 5: Statistical analysis for Supplemental Fig. 5A, Supplemental Fig. 5B, Supplemental Fig. 5C

One-way ANOVA of Log(D)						
**Source**	**Sum of squares**	**Degrees of freedom**	**F-statistic**	**PR(>F)**		
**Between Groups**	192545.603	7	14848.74759	<0.0001		
**Within Groups**	508429.9539	274464				
**Multiple Comparison of Means Log(D) — Tukey HSD, FWER=0.02**						
**Group 1**	**Group 2**	**MeanDiff**	**p-adj**	**lower**	**upper**	**reject**
ICP4 + dox (40 Hz)	ICP4 untreated (40 Hz)	-0.1737	<0.0001	-0.1997	-0.1478	True
ICP4 + dox (4 Hz)	ICP4 untreated (4 Hz)	-0.2773	<0.0001	-0.3563	-0.1984	True
ICP4 + dox (4 Hz)	ICP4 + dox (40 Hz)	-2.7826	<0.0001	-2.8375	-2.7276	True
**One-way ANOVA of α (alpha)**						
**Source**	**Sum of squares**	**Degrees of freedom**	**F-statistic**	**PR(>F)**		
**Between Groups**	272.8710557	7	218.0716408	<0.0001		
**Within Groups**	49062.04292	274464				
**Multiple Comparison of Means α (alpha) — Tukey HSD, FWER=0.02**						
**Group 1**	**Group 2**	**MeanDiff**	**p-adj**	**lower**	**upper**	**reject**
ICP4 + dox (40 Hz)	ICP4 untreated (40 Hz)	-0.0497	<0.0001	-0.0578	-0.0416	True
ICP4 + dox (4 Hz)	ICP4 untreated (4 Hz)	-0.0394	<0.0001	-0.0639	-0.0149	True
ICP4 + dox (4 Hz)	ICP4 + dox (40 Hz)	-0.0225	<0.0001	-0.0396	-0.0054	True

Supplemental Table 6: Statistical analysis for Supplemental Fig. 6C, Supplemental Fig. 6D

One-way ANOVA of Log(D_{100ms})						
**Source**	**Sum of squares**	**Degrees of freedom**	**F-statistic**	**PR(>F)**		
**Between Groups**	20844.7343	5	4703.52299	<0.0001		
**Within Groups**	347675.262	392257				
**Multiple Comparison of Means Log(D_{100ms}) — Tukey HSD, FWER=0.02**						
**Group 1**	**Group 2**	**MeanDiff**	**p-adj**	**lower**	**upper**	**reject**
HSV-1	Uninfected	-0.3986	<0.0001	-0.4148	-0.3824	True
HSV-1	HSV-1 (confluent)	-0.0718	<0.0001	-0.0861	-0.0574	True
HSV-1	HSV-1 + sorbitol	-0.333	<0.0001	-0.3484	-0.3175	True
**One-way ANOVA of α (alpha)**						
**Source**	**Sum of squares**	**Degrees of freedom**	**F-statistic**	**PR(>F)**		
**Between Groups**	626.433001	5	968.651285	<0.0001		
**Within Groups**	50735.0237	392257				
**Multiple Comparison of Means α (alpha) — Tukey HSD, FWER=0.02**						
**Group 1**	**Group 2**	**MeanDiff**	**p-adj**	**lower**	**upper**	**reject**
HSV-1	Uninfected	-0.0896	<0.0001	-0.0958	-0.0834	True
HSV-1	HSV-1 (confluent)	0.0038	0.2198	-0.0017	0.0093	False
HSV-1	HSV-1 + sorbitol	-0.036	<0.0001	-0.0419	-0.0301	True
